# Acetylcholinesterase inhibitory activity of sesquiterpenoids isolated from *Laggera pterodonta*

**DOI:** 10.3389/fpls.2023.1074184

**Published:** 2023-02-10

**Authors:** Jinliang Li, Fengchao Li, Guoxing Wu, Furong Gui, Hongmei Li, Lili Xu, Xiaojiang Hao, Yuhan Zhao, Xiao Ding, Xiaoping Qin

**Affiliations:** ^1^ State Key Laboratory for Conservation and Utilization of Bio-Resources in Yunnan, College of Plant Protection, Yunnan Agricultural University, Kunming, China; ^2^ State Key Laboratory of Phytochemistry and Plant Resources in West China, Kunming Institute of Botany, Chinese Academy of Sciences, Kunming, China; ^3^ College of Water Conservancy, Yunnan Agricultural University, Kunming, China

**Keywords:** *Laggera pterodonta*, sesquiterpenes, acetylcholinesterase, enzyme kinetics, toxic effects

## Abstract

Plant-derived natural products are important resources for pesticide discovery. Acetylcholinesterase (AChE) is a well-validated pesticide target, and inhibiting AChE proves fatal for insects. Recent studies have shown that the potential of various sesquiterpenoids as AChE inhibitors. However, few studies have been conducted with eudesmane-type sesquiterpenes with AChE inhibitory effects. Therefore, in this research, we isolated two new sesquiterpenes, laggeranines A (1) and B (2), along with six known eudesmane-type sesquiterpenes (3–8) from *Laggera pterodonta*, and characterized their structures and the inhibitory effect they exerted on AChE. The results showed that these compounds had certain inhibitory effects on AChE in a dose-dependent manner, of which compound 5 had the best inhibitory effect with IC50 of 437.33 ± 8.33 mM. As revealed by the Lineweaver-Burk and Dixon plots, compound 5 was observed to suppress AChE activity reversibly and competitively. Furthermore, all compounds exhibited certain toxicity levels on *C. elegans*. Meanwhile, these compounds had good ADMET properties. These results are significant for the discovery of new AChE targeting compounds, and also enrich the bioactivity activity repertoire of *L. pterodonta*.

## 1 Introduction

Acetylcholinesterase (AChE) is a critical enzyme performing important functions associated with nerve conduction, involving the catalysis of the degradation process of neurotransmitter acetylcholine and the subsequent termination of its stimulating effect on post-synaptic membrane excitation, through which the enzyme maintains normal nerve impulse transmission in organisms (Fournier and Mutero, 1994). In recent years, AChE has been studied in medicine, chemistry, pesticide and plant protection.

In pest control, AChE is a well-validated pesticide target, and inhibiting AChE proves fatal for insects (Rajashekar et al., 2014). Organophosphorus and carbamate insecticides are the most common AChE inhibitors (Fukuto, 1990). Although they play a great role in pest control, they can also cause harm to non-target organisms such as humans (Vale, 2015). In addition, due to the long-term use of the same insecticide, pests tend to develop resistance and make the inhibitors ineffective (Vaughan et al., 1997). So, we need to find new inhibitors to solve these problems. Many studies have shown that plant secondary metabolites are the main source of AChE inhibitors. In our previous study, 15 flavonoids isolated from *Eupatorium adenophorum* were discovered to suppress AChE in *Spodoptera litura* and *C. elegans* (Li et al., 2020), and 13 flavonoids isolated from *Ginkgo biloba* were found to inhibit AChE (Ding et al., 2013). Furthermore, we screened more than 200 compounds and the two sesquiterpenoids, parthenolide and tirotundin, extracted from *Chrysanthemum parthenium* and *Tithonia diversifolia*, respectively, elicited a strong inhibitory effect on nematode AChE (Lan et al., 2022). We think this work can provide some ideas for finding new inhibitors from natural products.

*Laggera pterodonta* (DC.) Benth. which grows in India, the Indochina Peninsula and tropical Africa (Gu et al., 2014), also extensively exist in southwestern China, particularly the Sichuan and Yunnan Provinces. This plant has long been used as folk medicine in China, and has been widely used clinically, with antioxidant, anti-tumor, antibacterial and analgesic effects. Previous studies on its constituents revealed that eudesmane-type sesquiterpene are one of the main secondary metabolites of this plant (Zhao et al., 1997; Yang et al., 2007; Lu et al., 2014; Xie et al., 2021). Sesquiterpenes are an important class of terpenoids with extensive biological activities, which have attracted our attention. A survey conducted by researchers found that 58 sesquiterpenes from various plants showed varying degrees of inhibitory activity against AChE in multiple studies over the past decade, and these findings shed light on the potential of sesquiterpenes to inhibit AChE (Arya et al., 2021). Therefore, we studied the chemical constituents of *L. pterodonta* and took the eudesmane-type sesquiterpene as the research object, trying to find the active compounds that inhibit AChE.

In this research, we isolated two new sesquiterpenes, laggeranines A (1) and B (2), along with six known sesquiterpenes (3–8), and characterized their structures and the inhibitory effect they exerted on AChE. At the same time, in order to understand the mechanism by which these compounds inhibit AChE, only compounds with strong activity levels were selected for kinetic studies. In addition, ADMET prediction is very important for early drug research and development. We also used the admeatSAR platform to make ADMET prediction for these compounds. This study provides experimental and theoretical foundations for novel acetylcholinesterase inhibitors in *L. pterodonta.*

## 2 Materials and methods

### 2.1 General experimental procedures

This study obtained chlorpyrifos (≥98% purity) from Sigma Chemical Co. (St. Louis, MO, USA), 5,5′-dithiobis-2-nitrobenzoic acid (DTNB, ≥98% purity) from Biological Engineering Co. (Huzhou, Zhejiang, China) and acetylcholine iodide (ATChI) (≥98% purity) from Fluka Chemical Co. (Milwaukee, WI, USA). Ultra-pure water (Milli-Q purification system, Millipore, MA) and acetonitrile (HPLC-grade, J.T. Baker, Phillipsburg, NJ) were utilized in semi-preparative HPLC. Additionally, petroleum ether, ethanol, acetone, ethyl acetate, methanol (MeOH), and chloroform of reagent grade were provided by Qingdao Marine Chemical Inc., China.

Bruker 500 and 600 MHz spectrometers were used to measure NMR spectra, using TMS as the endogenous reference. The BioRad FTS-135 spectrometer was utilized to survey IR spectra using KBr pellets, JASCO P-1020 digital polarimeter was employed for analyzing optical rotations, and the Shimadzu UV-2401A for recording UV spectra. The HR-ESI-MS were recorded on a triple quadrupole mass spectromete (Agilent, America). Furthermore, this study utilized silica gel (60–80, 200–300 and 300–400 mesh, Qingdao Marine Chemical Inc, China), SBC MCI gel (75–150 µm, Sci-Bio Chem Co. Ltd., Chengdu, China), Sephadex LH-20 (40–70 µm, Amersham Pharmacia Biotech AB) and silica gel H (10–40 µm, Qingdao Marine Chemical Inc, China) for Column Chromatography (CC). The YMC Luna C_18_ reversed-phase column (5 µm; 10 × 250 mm) was utilized for semi-preparative HPLC.

### 2.2 Plant material

For this study, aerial parts of *L. pterodonta* were collected in July 2017 from Baoshan, Yunnan, China (25°5′N, 99°6′E). Prof. Hua Peng from the Kunming Institute of Botany, Chinese Academy of Sciences (CAS), identified each of our collected samples. Meanwhile, we deposited one voucher specimen (No. 1707016) at the State Key Laboratory of Phytochemistry and Plant Resource in West China, Kunming Institute of Botany, CAS.

### 2.3 Extraction and isolation

Ethanol was added to the extract, containing dried aerial *L. pterodonta* (5 kg), thrice under room temperature (RT), followed by solvent evaporation in a vacuum. The obtained products were then filtered and evaporated to obtain 8 L extracts, which were divided, using equivalent amounts of ethyl acetate and petroleum ether (thrice with each), to obtain ethyl acetate extracts (55 g) and petroleum ether extracts (68 g). Later, a silica gel column (10 × 100 cm) was used for the chromatography of EtOAc extracts and eluted using petroleum ether-acetone (100:1-1:1) to obtain 8 fractions (1–8).

MCI chromatography was conducted to purify fraction 3 (278 mg) and eluted using the MeOH–H2O mixed solution, which generated 4 fractions (3A–3D). We utilized silica gel to purify fraction 3B (102 mg) using petroleum ether-ethyl acetate (50:1–10:1), generating Fr.3B2 (14 mg), which was subsequently purified by semi-preparative HPLC (54% CH3CN within the water) to yield compound 1 (3.4 mg, tR = 15 min).

MCI chromatography was also utilized to purify fraction 4 (45.6 g), which was eluted using the MeOH-H2O mixed solution to yield eight corresponding fractions (4A–4H). Of these, we purified fraction 4D (10.5 g) using the silica gel column and performed the elution using petroleum ether-ethyl acetate (40:1–4:1, stepwise) to obtain another 5 fractions (4D1–4D5). Sephadex LH-20 CC was performed for the chromatography of Fr.4D2 (3.9 g) under MeOH elution, followed by semi-preparative HPLC, using the 58% acetonitrile solvent system (3 mL/min), to obtain compound 3 (226 mg, tR = 55 min), compound 4 (1.9 g, tR = 59 min) and compound 5 (213 mg, tR = 65 min). Similarly, we used the silica gel column to purify fraction 4E (221 mg), and elution was performed using petroleum ether-ethyl acetate (70:1–4:1, stepwise) to obtain three further fractions (4E1–4E3). Later, Sephadex LH-20 CC was employed to purify Fr.4E2 (46 mg), fraction 4F (2.1 g), and fraction 4G (104 mg) under MeOH elution, followed by semi-preparative HPLC, using the 55%, 47% and 45% acetonitrile solvent systems (3 mL/min), respectively, to yield compound 7 (10.5 mg, tR = 60 min), compound 6 (1.3 g) and compound 8 (76 mg, tR = 19 min), respectively.

The silica gel column was then utilized to purify fraction 7 (708 mg) and eluted using petroleum ether-ethyl acetate (40:1–5:1, stepwise) to generate fractions 7A-7G. Sephadex LH-20 CC was then performed to purify fraction 7C (56 mg) using a mobile phase of [dichloromethane-methanol (1:1)], which yielded two fractions (7C1–7C2). Semipreparative HPLC was adopted to purify Fr.7C2 (15 mg) with the 57% acetonitrile solvent system (3 mL/min) to yield compound 2 (2.8 mg, tR 45 min).

#### 2.3.1 laggeranine A (1)

${\left[\alpha \right]}_{\text{D}}^{20}-159.13$ (*c* 0.16, MeOH); UV (MeOH) Λ_max_ (log ε): 195 (4.17) nm; IR (KBr): 3429, 2930, 2874, 1695, 1623, 1461, 1434, 1384, 1264, 1189, 1151, 1081 cm^-1^; ^1^H and ^13^C NMR data, see **Table 1**. HR-ESI-MS: *m/z* 249.1495 [M-H]^-^ (calcd for C_15_H_22_O_3_, 249.1496).

**Table 1:** ^1^H and ^13^C NMR Data of Compounds 1 and 2 (δ in ppm, J in Hz, 600 MHz for 1H and 150 MHz for 13C, in Methanol-d4).

Position	1		2	
	δ_H_ (*J* in Hz)	δ_C,_ type	δ_H_ (*J* in Hz)	δ_C,_ type
1a	5.50, d (4.8)	123.9, CH	1.89, td (4.2, 13.8)	37.8, CH_2_
1b			1.18, d (13.8)	
2a	4.00, m	65.1, CH	1.51, m	23.2, CH_2_
2b			1.61, m	
3a	1.56, dt (1.5, 14.4)	37.5, CH_2_	2.14, d (13.8)	34.2, CH_2_
3b	1.69, m		2.52, td (4.8, 13.8)	
4	1.90, m	32.4, CH	–	150.8, C
5	–	39.9, C	–	76.2, C
6a	1.62, dd (9.7, 13.4)	41.8, CH_2_	1.57, m	39.0, CH_2_
6b	1.68, m		2.09, d (12.8)	
7	2.64, m	34.3, CH	2.87, m	38.1, CH
8a	1.74, m	31.1, CH_2_	1.52, m	27.4, CH_2_
8b	1.83, m		1.67, ddd (3.3, 13.2, 25.8)	
9a	2.05, ddd (2.2, 8.1, 13.7)	29.2, CH_2_	1.82, td (3.3, 13.2)	34.5, CH_2_
9b	2.49, m		1.10, d (13.2)	
10	–	149.8, C	–	39.7, C
11	–	148.0, C	–	147.0, C
12	–	171.6, C	–	168.8, C
13a	5.56, br. s	122.5, CH_2_	5.61, br. s	123.1, CH_2_
13b	6.09, br. s		6.14, br. s	
14a	0.87, d (6.8)	16.1, CH_3_	4.97, br. s	111.7 CH_2_
14b			5.26, br. s	
15	0.88, s	19.6, CH_3_	1.04, s	23.1, CH_3_
16	–	–	4.21, m	61.8, CH_2_
17	–	–	1.30, t (6.0)	14.5, CH_3_

The NMR signal abbreviations:br, broad; s, singlet; d, doublet; t, triplet; m, multiplet; dt, doublet of triplets; td, triplet of doublet; dd, doublet of doublets; ddd, doublet of dd. "–" represents quaternary carbon.

#### 2.3.2 laggeranine B (2)

${\left[\alpha \right]}_{\text{D}}^{20}$ − 4.76 (*c* 0.14, MeOH); UV (MeOH) Λ_max_ (log ε): 195 (3.64) nm; IR (KBr): 3434, 2927, 2855, 1714, 1627, 1448, 1383, 1263, 1171, 1123, 1047 cm^-1^; ^1^H and ^13^C NMR data, see **Table 1**. HR-ESI-MS: *m/z* 301.1773 [M + Na] ^+^ (calcd for C_17_H_26_O_3_, 301.1774).

### 2.4 Nematode

This investigation acquired *C. elegans* from the Insect Toxicology Laboratory of Yunnan Agricultural University, Kunming, Yunnan, China, and cultivated them using an oat medium under RT.

### 2.5 Determination of IC_50_ of compounds to AChE

Third-instar juvenile stage samples of *C. elegans* were thoroughly ground using a glass homogenizer. The homogenate was then dissolved in PBS (pH 7.0, which contained 0.1% Triton X-100) using a suitable volume, followed by a 30-min centrifugation at 6000 r/min under 4°C to harvest supernatants to analyze the enzymes (Ellman et al., 1961; Zhao et al., 2018) [Nematode (3rd instar): 4000 individuals/mL].

After dissolving the eight compounds and chlorpyrifos in acetone, multiple dilutions were made to obtain test concentrations at 5 levels. Thereafter, the test solution of every level (4 µL) was blended with the enzyme solution (96 µL), followed by 2-h incubation at 37°C in a 96-well plate. The final concentrations of compounds were 29.25, 58.50, 117.00, 234.00, and 468.00 µg/mL, respectively. Thereafter, 1.5 mM acetylthiocholine iodide (ATCHI) (50 µL) was added for a further 0.5-h incubation at 37°C. The reaction was finally terminated by adding 0.3 mM 5,5′- dithiobis (2 - nitrobenzoic acid) (DTNB) (50 µL). Afterward, the residual activity of acetylcholinesterase was measured with a microplate reader at 405 nm. Corresponding treatments were then applied to different groups as given below:

1. Treatment group:

Enzyme solution+compound+ATCHI(1.5mM,50μL)+DTNB(0.3mM,50μL).

2. Compound control group:

Enzyme solution+compound+PBS(50μL)+PBS(50μL).

3. Substrate control group:

Enzyme solution+ATCHI(1.5mM,50μL)+DTNB(0.3mM,50μL).

4. PBS control group:

Enzyme solution+PBS(50μL)+PBS(50μL).

The inhibition rate (%) was calculated follows:


I%=[1−(Treatment group−Compound control group)/(Substrate control group−PBS control group)]×100%

### 2.6 Kinetic study on the inhibitory effect of AChE by compound

This study prepared the enzyme solution at multiple gradients (0.125, 0.25, 0.5, and 1.0 U/mL). After adding compound 5 (29.25, 58.50, 117.00, 234.00, and 468.00 µg/mL), the mixed sample was then subjected to a 1-h incubation on a 96-well plate at 37°C (the buffer was used as a substitute for the control group). Later, 1.5 mM ATCHI (50 µL) was added and incubated for a 30-min period, followed by an addition of the 0.3 mM DTNB (50 µL). After 30 s, a microplate reader was utilized to record the OD value at 405 nm (five times at 1-min intervals). The change in the OD value every minute was used to calculate the reaction rate (ΔA/min). Later, the reaction rate curve, as a function of enzyme concentration, was plotted to compare reaction rates (*v*) among diverse test compound levels. Associations between diverse enzyme levels were utilized to evaluate the inhibitory effect of the compound on nematode AChE (Xiong et al., 2016). In addition, the Lineweaver-Burk double reciprocal graph, demonstrating the reaction rate as a function of enzyme level, was plotted to infer the inhibition type (Guo et al., 2018).

### 2.7 Toxic effects of compounds on *C. elegans*

In the toxicity analysis, 95 µL of the nematode solution (containing approximately 80 C*. elegans*) along with 5 µL of each compound was added to a 96-well plate. The final concentration of each compound was 0.5 mg/mL, and chlorpyrifos (20 µg/mL) was used as the positive control. The samples were mixed sufficiently, followed by 48-h incubation of plates under RT, and subsequently, the dead nematodes were counted to determine the lethality rate (Roh and Choi, 2008).

### 2.8 ADMET prediction of compounds

Will compound SMILES format into admetSAR web site (http://lmmd.ecust.edu.cn/admetsar1) prediction module, the small molecule ADMET forecast information can be obtained by clicking on Predict.

### 2.9 Data analysis

GraphPad Prism 8 was employed for kinetics analysis and IC_50_ value determination of AChE, while SPSS18 was employed for calculating the statistical significance. Data in the bar graph are represented as the mean ± SD. Duncan’s new multiple range analysis was conducted to compare and analyze the significance of the difference, where *p<*0.05 indicated for a significant difference. Each experiment was conducted in three independent replicates.

## 3 Results and discussion

### 3.1 Structural elucidation of compounds 1-8

**Figure 1:**
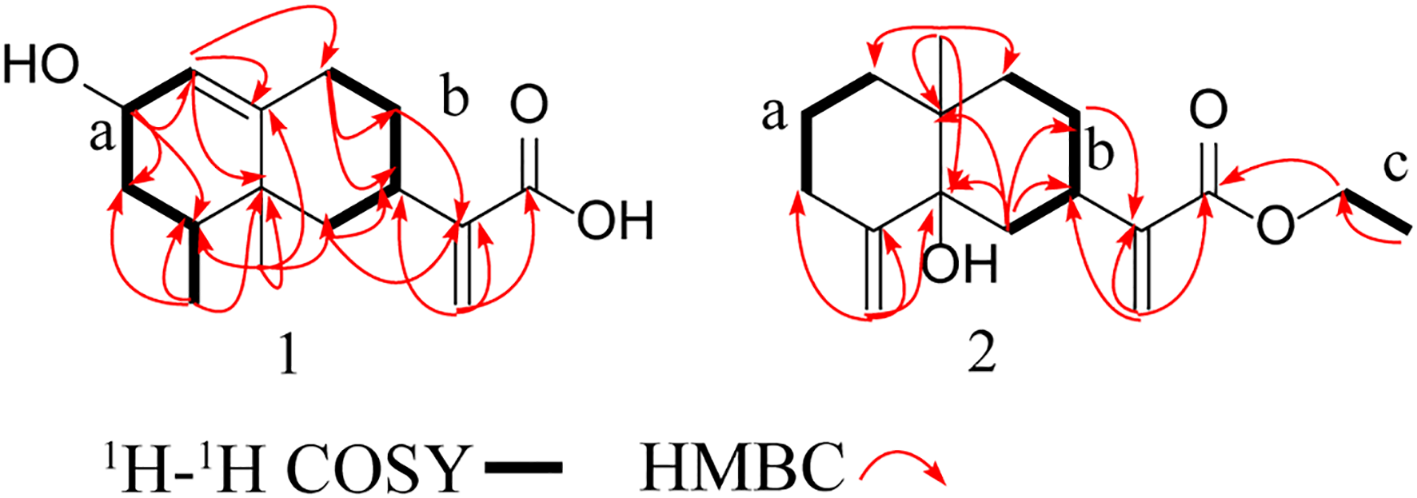
Key HMBC, 1 H-1 H COSY, and HMBC correlations of compounds 1 and 2.

This study identified compound 1 as a colorless oily substance, whose molecular formula was determined to be C_15_H_22_O_3_ based on HR-ESI-MS at *m/z* 249.1495 ([M-H] ^−^, calcd, 249.1496). Besides, its IR absorption bands were detected at 3429 cm^–1^ and 1695 cm^–1^, implying the presence of one conjugated carboxylic acid group. As observed from the ^1^H NMR spectrum (**Table 1**), there were signals for three olefinic protons (δ_H_ 5.50, 5.56, and 6.09) and two methyls groups (δ_H_ 0.87, 0.88). Both DEPT and ^13^C NMR spectroscopy analyses (**Table 1**) identified 15 carbon signals, which included two methyl (δ_C_ 16.1 and 19.6), five methylenes (δ_C_ 29.2, 31.1, 37.5, 41.8, and 122.5), four methines (δ_C_ 32.4, 34.3, 65.1, and 123.9) and four quaternary carbons (δ_C_ 39.9, 148.0, 149.8, and 171.6). Correlations in the ^1^H-^1^H HSQC and COSY diagrams suggested that there were 2 proton-bearing fragments, CHCHCH_2_CHMe (a) and CH_2_CHCH_2_CH_2_ (b) (**Figure 1**). In the HMBC spectrum, 2 methyl groups [δ_H_ 0.87 (3H, d, *J =* 6.8 Hz) and δ_H_ 0.88 (3H, s)] exhibited HMBC associations with C-4 and C-5, which indicated their positions within nearby carbons. Meanwhile, the HMBC associations between H_3–_14 and C-3 as well as H_3–_15 with C-6 revealed the C-14/C-4/C-5(C-15)/C-6 association. Besides, HMBC associations between H_3–_15 and C-10; H-1 and C-9, C-10; H-9 and C-7, C-8, revealed the C-1/C-10(C-5)/C-9 connection. Therefore, as per the associations of H-6 with C-7 and C-11, and H-13 with C-7, C-11, as well as C-12, we determined the compound skeleton. (**Figure 1**).

**Figure 2:**
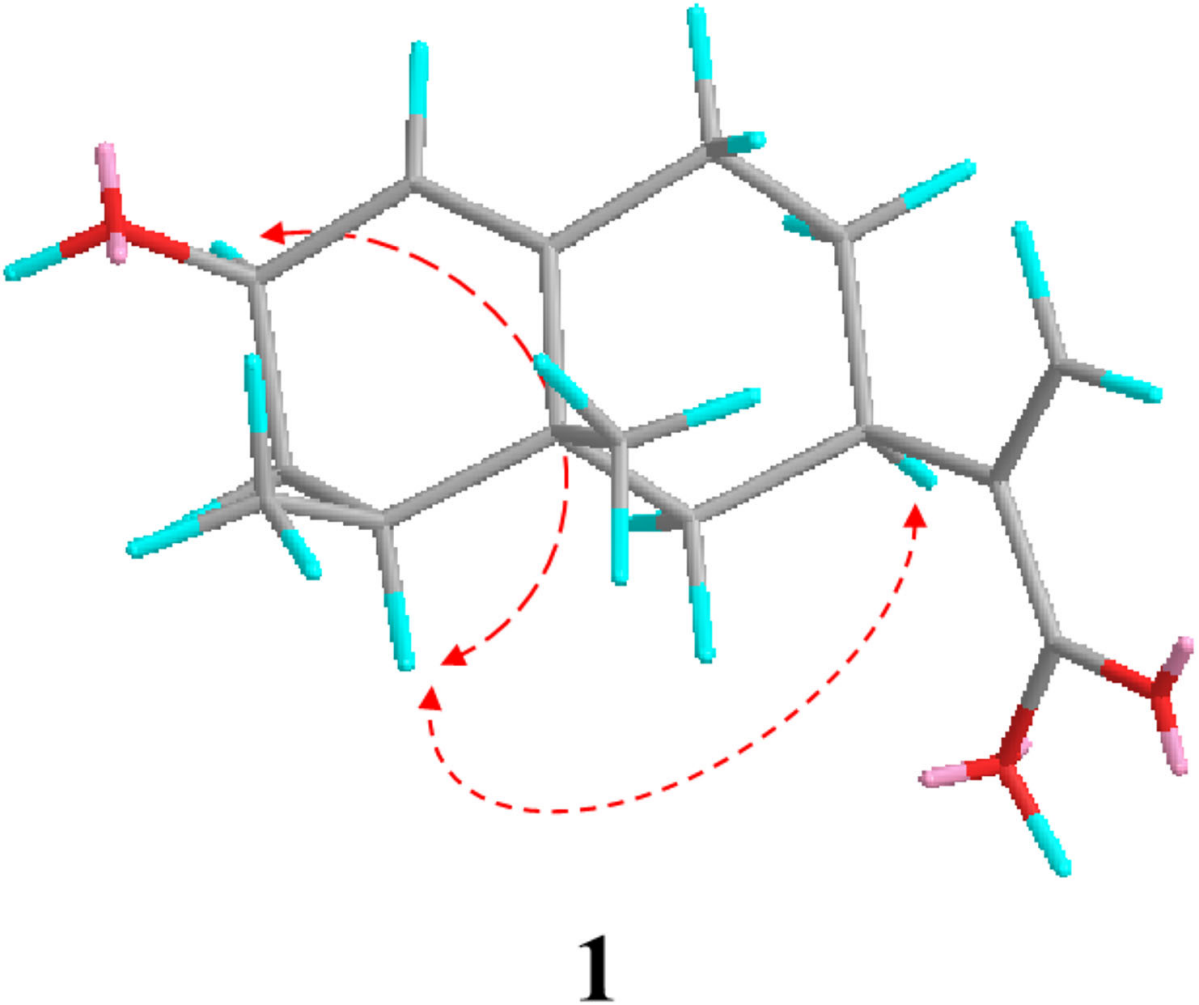
Key ROESY correlations of compound 1.

**Figure 3:**
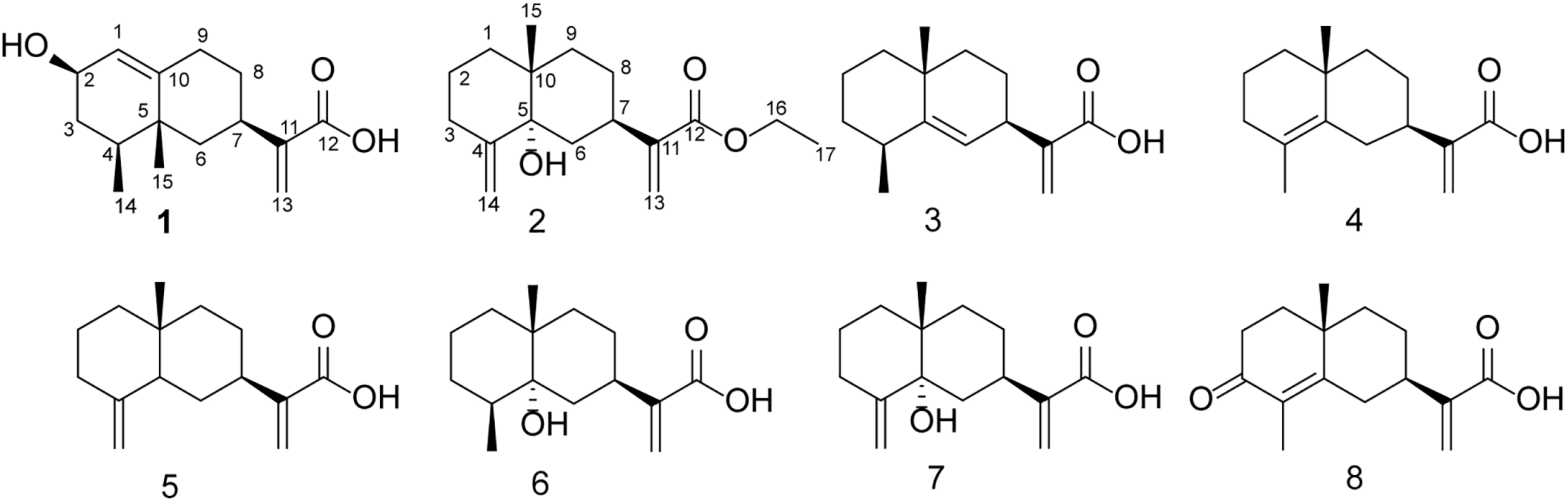
Chemical structures of the eight compounds.

As indicated by further HMBC analyses, 1 had a close structural resemblance to tessaric acid, a known eremophilane sesquiterpene (Marcela et al., 1997). The only major discrepancy was the replacement of C=0 group of tessaric acid, with a hydroxyl group of 1, which was inferred from H-1, H-3, H-4 with C-2 cross-peaks in HMBC. The ROESY spectrum (**Figure 2**) was used to assign the relative configuration for 1, where H-2/H-4 and H-4/H-7 associations suggested that they were co-facial with an α-orientation. Since 1 had a specific rotation ( ${\left[\alpha \right]}_{\text{D}}^{20}$ –159.1), similar to that of tessaric acid ( ${\left[\alpha \right]}_{\text{D}}^{20}$ –156.2). The specific rotation of tessaric acid was determined by X-ray single-crystal diffraction (Zheng et al., 2003), and the absolute configuration of compound 1 was be the same as that of tessaric acid. Therefore, the structure of compound 1 could be inferred through the above rationale (**Figure 3**).

Compound 2 was also identified as a colorless oily substance. Based on HR-ESI-MS at *m/z* 301.1773 ([M + Na] ^+^, calcd, 301.1774), we determined the molecular formula to be C_17_H_26_O_3_. As revealed by the IR spectrum, the typical absorption bands were observed for hydroxy (3434 cm^−1^) and ester carbonyl (1714 cm^−1^) functional groups. Signals for four olefinic protons (δ_H_ 4.97, 5.26, 5.61, and 6.14) and two methyls (δ_H_ 1.04 and 1.30) were observed from the ^1^H NMR spectrum (**Table 1**). Besides, 17 carbon signals, which included 2 methyl (δ_C_ 14.5, 23.1), nine methylenes (δ_C_ 23.2, 27.4, 34.2, 34.5, 37.8, 39.0, 61.8, 111.7, and 123.1), one methine (δ_C_ 38.0) and five quaternary carbons (δ_C_ 39.7, 76.2, 147.0, 150.8, and 168.8), were observed from DEPT and ^13^C NMR spectroscopy (**Table 1**). Besides, ^1^H along with ^13^C NMR spectroscopy for 2 (**Table 1**) displayed close resemblance to 5α-hydroxycostic acid (Sanz et al., 1990), but with an additional C_2_H_5_O group (δ_C_ 61.8 and δ_C_ 14.5). 2D NMR data was utilized for subsequent analysis. According to ^1^H-^1^H COSY associations, 3 fragments: a (C-1–C-3), b (C-8/C-9), and c (C-16-C17), were present (**Figure 1**). Moreover, the C_2_H_5_O group existed in carboxyl (C-12), according to the HMBC association of H-13 with C-7, C-11, C-12; H_3–_17 with C-16, as well as H-16 with C-12. The structure of 2 was thereby characterized this way (**Figure 3**).

For known compounds, ^1^H NMR and ^13^C NMR spectra were determined and compared with the data in the literature, the structures were determined to be eudesma-5,12-dien-13-oic acid (3) (Xu et al., 2006), isocostic acid (4) (Cruz and Martinez, 1982), costic acid (5) (Bawdekar and Kelkar, 1965), 5α-hydroxy-4α,15-dihydrocostic acid (6) (Xie et al., 2011), 5α-hydroxycostic acid (7) (Xie et al., 2011), and 3-oxo-di-nor-eudesma-4-en-11-oic acid (8) (Wang et al., 2013). ^1^H NMR and ^13^C NMR spectra can be found in supporting materials.

### 3.2 Determination of IC_50_ of compounds to AChE

**Figure 4:**
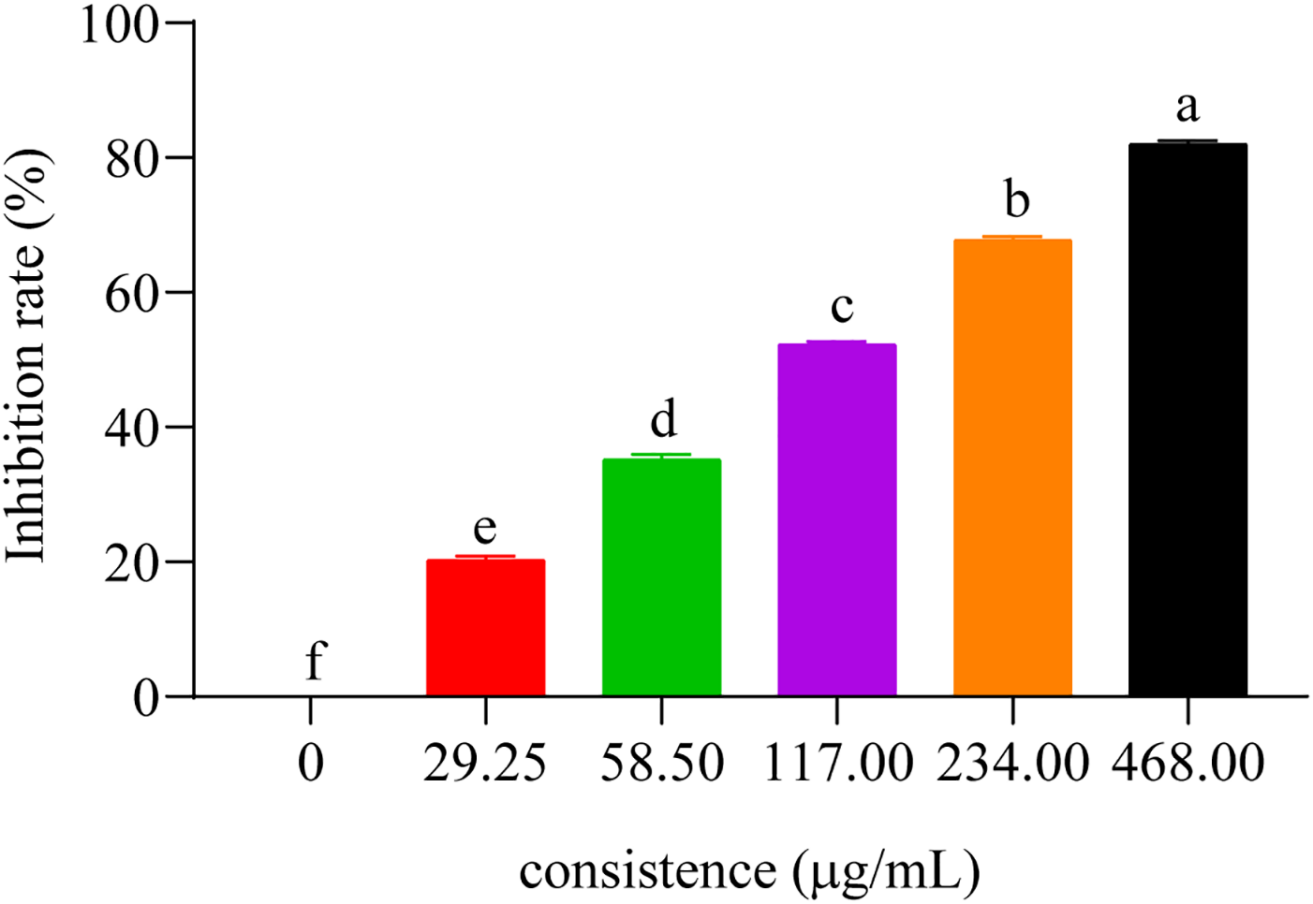
The inhibitory effect of compound 5 on the AChE. Concentrations of compound 5 (29.25, 58.50, 117.00, 234.00, and 468.00 μg/mL) for curves are indicated in the column.

To evaluate the inhibitory activity of these compounds on AChE activity, we measured their IC_50_ values with respect to *C. elegans* AChE (chlorpyrifos being utilized as the positive control). IC_50_ represents the compound dose required for 50% inhibition of AChE activity, where a lower value would indicate a more potent inhibition on AChE (Wang et al., 2021). The results showed that those eight compounds inhibited *C. elegans* AChE in a dose-dependent manner, such as compound 5 (**Figure 4**), and the most effective inhibitory effects were compound 5 (IC_50_ = 437.33 ± 8.33 μM), followed by compound 3 (IC_50_ = 464.0 ± 14.74 μM), compound 6 (IC_50_ = 470.33 ± 15.57 μM), compound 7 (IC_50_ = 485.0 ± 11.53 μM), compound 1, compound 4 (IC_50_ < 530 μM), and compounds 2 and 8 (IC_50_ < 710 μM) (**Table 2**).

**Table 2:** IC_50_ of AChE inhibition by compounds.

Compounds	IC_50_ (μM)
1	518.33 ± 5.90^b^
2	701.33 ± 14.74^a^
3	464.00 ± 10.00^d^
4	527.67 ± 8.96^b^
5	437.33 ± 8.33^e^
6	470.33 ± 15.57^cd^
7	485.00 ± 11.53^c^
8	696.67 ± 12.42^a^
Chlorpyrifos	7.33 ± 0.58^f^

The effects of the tested compounds on AChE were repeated in triplicates; IC_50_ values represent the means ± SD; different letters indicate significant differences (p< 0.05).

Among the 8 compounds, compound 2 showed the worst inhibitory activity, and except compound 2, all the other compounds were eudesmane-type sesquiterpene acids. Therefore, we hypothesized that the carboxyl group in the compound should increase the activity. Compounds 3, 4, and 5 are isomers, while compound 5 shows better activity. Before this, the cytotoxic activity of compounds 4 and 5 against SF9 was also reported, and the same compound 5 showed better activity (Azucena et al., 2005). The difference in their structure lies in the different positions of a double bond. Therefore, we believe that the change of the position of the double bond has certain influence on the activity, and the terminal double bond of the compound should have better activity. The only structural difference between compound 4 and compound 8 is that the C-3 of compound 8 is a carbonyl group, but the activity of compound 4 is significantly higher than that of compound 8. In addition, we found that the hydroxyl group is connected to the skeletons of compounds 1, 6 and 7, but their activities are also not ideal. Can the presence of hydroxyl and carbonyl groups on the eudesmane-type sesquiterpene acid skeleton reduce the inhibitory activity of the compound against AChE? For this purpose, we reviewed studies in the last decade on the inhibition of AChE activity by sesquiterpenoids. However, prior to this study, there were no studies on the inhibition of AChE by the eudesmane-type sesquiterpene acid. Therefore, we hypothesized that the addition of hydroxyl or carbonyl groups to the backbone of eudesmane-type sesquiterpene acid may weaken the inhibitory effect of the compounds on AChE, but this needs to be verified by more experiments.

Compared with various sesquiterpenoids with acetylcholinesterase inhibitory activities reported in the literature, 8 compounds in this study showed unsatisfactory inhibitory activities. Meanwhile, we found that other eudesmane-type sesquiterpenes and their derivatives reported in the literature also showed poor inhibitory activity against AChE, while among the various constituents with AChE inhibitory activity, various sesquiterpene lactones seemed to be the most promising AChE inhibitors.

### 3.3 Kinetic study on the inhibitory effect of AChE by compound

**Figure 5:**
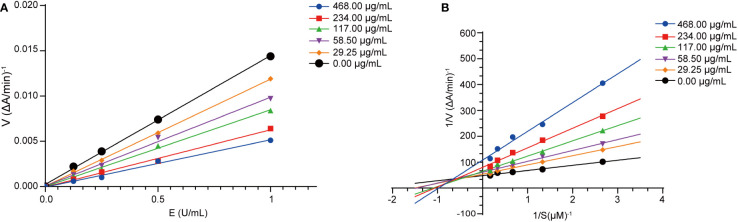
Acetylcholinesterase inhibition kinetics analysis of compound 5. **(A)** Hydrolytic activity of acetylcholinesterase concentration under the action of different concentrations of compound 5 (29.25, 58.50, 117.00, 234.00, and 468.00 μg/mL). **(B)** Lineweaver-Burk plots for the inhibition of compound 5 (29.25, 58.50, 117.00, 234.00, and 468.00 μg/mL).

To further explore the inhibition mechanism against AChE, we conducted a kinetics analysis on compound 5. The relationship between the maximal reaction initial speed (*v*) and diverse enzyme levels was analyzed by evaluating the reversible inhibitory effect exerted by compound 5 (**Figure 5A**). According to **Figure 5A**, the fitting curve indicated the original rate under diverse levels of compound 5 and a straight line was observed for the enzyme level. Moreover, every straight line intersected at the origin, with the slope dropping as the compound level increased, implying that compound 5 was a reversible inhibitor of AChE.

Under the scenario of compound 5 being identified as a reversible inhibitor, we adopted the double-reciprocal graph (Lineweaver-Burk) to model the maximal original velocity and substrate level to explore the inhibition patterns. According to **Figure 5B**, each straight line had a unique intercept and slope but intersected within the second quadrant in the Lineweaver-Burk diagram. Meanwhile, as the compound level increased, fitted curves had elevated X-intercepts (-1/Km), Y-intercept (1/Vmax) and slopes, which indicated an increase in the Michaelis constant Km, but a decrease in Vmax. Consequently, compound 5 inhibited AChE in a mixed-type competitive manner, and could be an AChE inhibitor with dual binding sites (Shaik et al., 2019; Tang et al., 2019).

**Figure 6:**
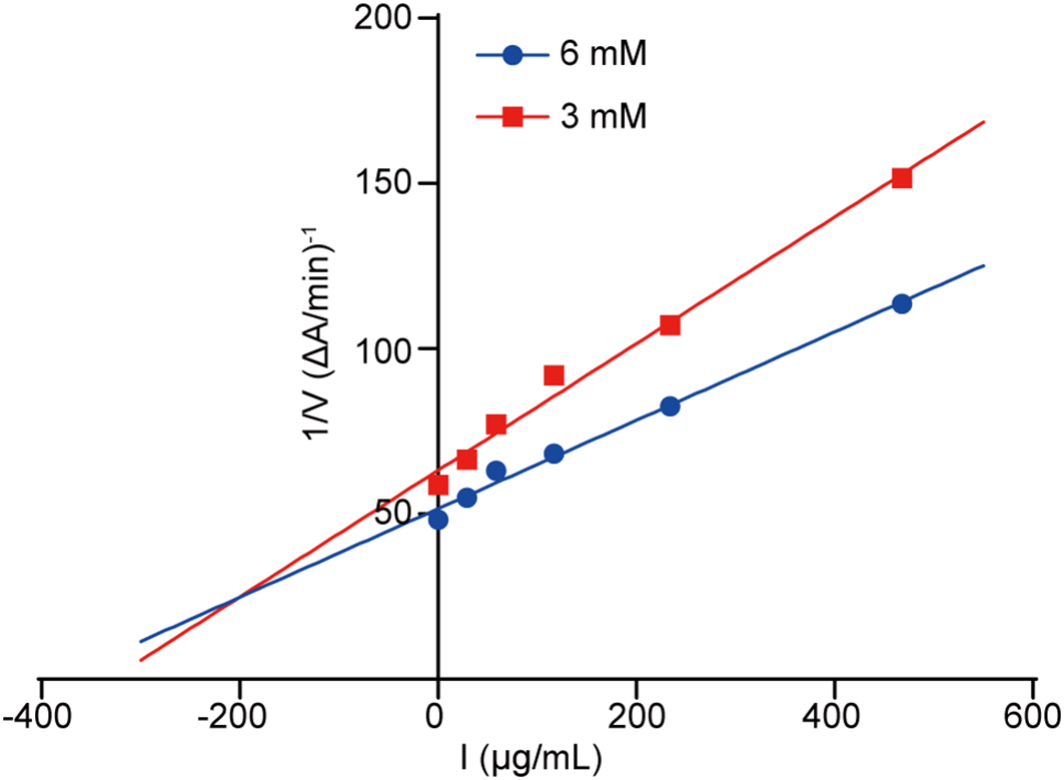
Dixon diagram. Dixon plot showing the inhibitory activities of compound 5 against AChE in the presence of different concentrations of substrate. Red and blue lines indicate concentrations of 3 and 6 mM, respectively.

The Dixon plot was made to ascertain the AChE inhibition pattern as well as the dissociation constant (Ki) of our tested compounds. Typically, the Ki value represents the dissociation constant of the enzyme-inhibitor complex, where a lower value indicates a higher affinity of the compound to AChE. Based on the Dixon plot (**Figure 6**) and the slope in the double reciprocal graph (regarding compound 5 level with maximal original velocity), compound 5 had a Ki value of 24.668 µg/mL (**Figure 6**).

### 3.4 Toxic effects of compounds on *C. elegan*s

To evaluate the level of toxicity on *C. elegans*, we chose eight compounds for analysis. According to the results, the compounds had a potential toxicity level with respect to *C. elegans* at the experimental concentration, where compounds 3 and 5 were highly toxic, with median lethality rates of 60.83 ± 2.6% and 72.92 ± 3.15%, respectively (**Table 3**). Similarly, compound 2 still showed the lowest toxicity level, which further proved that the carboxyl group in the compound could increase the activity. Compounds 3, 4, and 5 as isomers also showed different toxicity levels, which further confirmed that the change of double bond position had a certain effect on the activity, and the terminal double bond of the compound should have a better activity. At the same time, the toxicity of compounds 1, 6, 7, and 8 with hydroxyl or carbonyl groups in the skeleton is still not ideal, which increases the possibility that the addition of hydroxyl or carbonyl groups to the main chain of the eudesmane-type sesquiterpene acid may reduce the toxicity of the compounds.

**Table 3:** The lethality of eight compounds to *C. elegans*.

Compounds	Lethality rate (%)
1	38.75 ± 2.50^d^
2	31.67 ± 0.72^e^
3	60.83 ± 2.60^b^
4	30.83 ± 1.91^e^
5	72.92 ± 3.15^a^
6	48.75 ± 2.50^c^
7	50.83 ± 1.91^c^
8	34.58 ± 1.44^e^
Chlorpyrifos	81.67 ± 2.60^f^

The effects of the tested compounds on C. elegans were repeated in triplicates; the values represent the means ± SD; different letters indicate significant differences (p< 0.05).

The *in vivo* toxicological results of these compounds were similar to those of AChE inhibition experiments, suggesting that the toxic effects of these compounds on *C. elegans* may be related to their inhibitory activity on AChE to some extent. However, this is not enough to conclude that there was a causal relationship between them. Previous studies have shown that drug toxicity to nematodes occurs in multiple biological tissues (Roh and Choi, 2008). To understand the relationship between AChE activity and toxicity, further experiments are needed for verification.

### 3.5 ADMET prediction of compounds

The main indexes of ADMET prediction were Blood Brain Barrier (BBB), Human Intestinal Absorption (HIA), Caco-2 permeability (CCP) and Ames mutagenesis (ATT), Carcinogenicity and cytochrome CYP2D6 are shown in **Table 4**. The 8 compounds isolated from *L. pterodonta* were easy to be absorbed or assimilated by the human intestine and could penetrate human intestinal cell lines without mutagenic toxicity or carcinogenicity. This indicates shown that these compounds have good ADMET properties. In addition, except for compounds 1, 6, and 7, which could not easily cross the blood-brain barrier, all other compounds could easily cross the blood-brain barrier. Comparing the structural differences between compounds 1, 6, and 7 and other compounds, it was found that this might be related to the hydroxyl group connected to the skeleton of these three compounds.

**Table 4:** ADMET prediction results for compounds.

Compound	BBB	HIA	CCP	ATT	Carcinogenicity	CYP2D6 inhibitor
1	0.5000−	0.9947+	0.8022+	None	None	non-inhibitor
2	0.7250+	1.000+	0.8954+	None	None	non-inhibitor
3	0.7000+	0.9946+	0.8665+	None	None	non-inhibitor
4	0.7000+	0.9946+	0.8382+	None	None	non-inhibitor
5	0.7250+	0.9950+	0.7249+	None	None	non-inhibitor
6	0.5000−	0.9956+	0.7609+	None	None	non-inhibitor
7	0.7250−	0.9959+	0.6820+	None	None	non-inhibitor
8	0.6500+	0.9929+	0.7555+	None	None	non-inhibitor

BBB “+” represents that drug molecules can easily cross the blood-brain barrier; BBB “−“ represents that drug molecules cannot easily cross the blood-brain barrier; a value closer to 1 indicates better permeability to BBB; HIA “+” represents that drug molecules can be absorbed or assimilated through the human intestine, and a value closer to 1 indicates better absorption through the intestine.;CCP “+” means that it can easily penetrate human intestinal cell lines, and the closer the value is to 1, the better CCP permeability is. “None” means that the compound has no mutagenic toxicity or carcinogenicity.

## 4 Conclusions

This study tested novel sesquiterpenes and six known eudesmane-type sesquiterpene acid isolated from *L. pterodonta*, to analyze their inhibitory activity on *C. elegans* AChE. The results showed that all these compounds had certain inhibitory effects on AChE in a dose-dependent manner, of which compound 5 had the best inhibitory effect with IC_50_ of 437.33 ± 8.33 μM. Meanwhile, as revealed by the Lineweaver-Burk and Dixon plots, compound 5 was observed to suppress AChE activity reversibly and competitively. Furthermore, all 8 compounds exhibited certain toxicity levels on *C. elegans*. Finally, the ADMET prediction was carried out for these 8 compounds, and it was found that all compounds had good ADMET properties. Collectively, we believe that these results are significant for the discovery of new AChE targeting compounds, and also enrich the bioactivity activity repertoire of *L. pterodonta.*

## Data availability statement

The raw data supporting the conclusions of this article will be made available by the authors, without undue reservation.

## Author contributions

JL, XQ, and XD conceived and designed the experiments. JL and FL performed the experiments and analyzed the data. XQ, XD, GW, FG, HL, LX, YZ and XH contributed reagents, materials, and analysis tools. All authors contributed to the article and approved the submitted version.
