# Supplementary material for: Acetylcholinesterase inhibitory activity of sesquiterpenoids isolated from Laggera pterodonta
Source: Front Plant Sci. 2023 Feb 10;14:1074184. doi: 10.3389/fpls.2023.1074184 (PMC9950556; doi:10.3389/fpls.2023.1074184)
Supplement: Supplementary file 1 [file DataSheet_1.docx]

Supplementary Material

**Figure S1** ^1^H NMR spectrum of laggeranine A in Methanol-*d*_4_

**Figure S2** ^13^C NMR spectrum of laggeranine A in Methanol-*d*_4_

**Figure S3** HSQC spectrum of laggeranine A in Methanol-*d*_4_

**Figure S4** ^1^H-^1^H COSY spectrum of laggeranine A in Methanol-*d*_4_

**Figure S5** HMBC spectrum of laggeranine A in Methanol-*d*_4_

**Figure S6** ROESY spectrum of laggeranine A in Methanol-*d*_4_

**Figure S7** HRESIMS spectrum of laggeranine A

**Figure S8** IR spectrum of laggeranine A

**Figure S9** UV spectrum of laggeranine A

**Figure S10** ^1^H NMR spectrum of laggeranine B in Methanol-*d*_4_

**Figure S11** ^13^C NMR spectrum of laggeranine B in Methanol-*d*_4_

**Figure S12** HSQC spectrum of laggeranine B in Methanol-*d*_4_

**Figure S13** ^1^H-^1^H COSY spectrum of laggeranine B in Methanol-*d*_4_

**Figure S14** HMBC spectrum of laggeranine B in Methanol- *d*_4_

**Figure S15** ROESY spectrum of laggeranine B in Methanol- *d*_4_

**Figure S16** HRESIMS spectrum of laggeranine B

**Figure S17** IR spectrum of laggeranine B

**Figure S18** UV spectrum of laggeranine B

**Figure S19** ^1^H NMR spectrum of compound **3** A in CDCl_3_

**Figure S20** ^13^C NMR spectrum of compound **3** in CDCl_3_

**Figure S21** ^1^H NMR spectrum of compound **4** in Methanol-*d*_4_

**Figure S22** ^13^C NMR spectrum of compound **4** in Methanol-*d*_4_

**Figure S23** ^1^H NMR spectrum of compound **5** in Methanol-*d*_4_

**Figure S24** ^13^C NMR spectrum of compound **5** in Methanol-*d*_4_

**Figure S25** ^1^H NMR spectrum of compound **6** in CDCl_3_

**Figure S26** ^13^C NMR spectrum of compound **6** in CDCl_3_

**Figure S27** ^1^H NMR spectrum of compound **7** in CDCl_3_

**Figure S28** ^13^C NMR spectrum of compound **7** in CDCl_3_

**Figure S29** ^1^H NMR spectrum of compound **8** in CDCl_3_

**Figure S30** ^13^C NMR spectrum of compound **8** in CDCl_3_


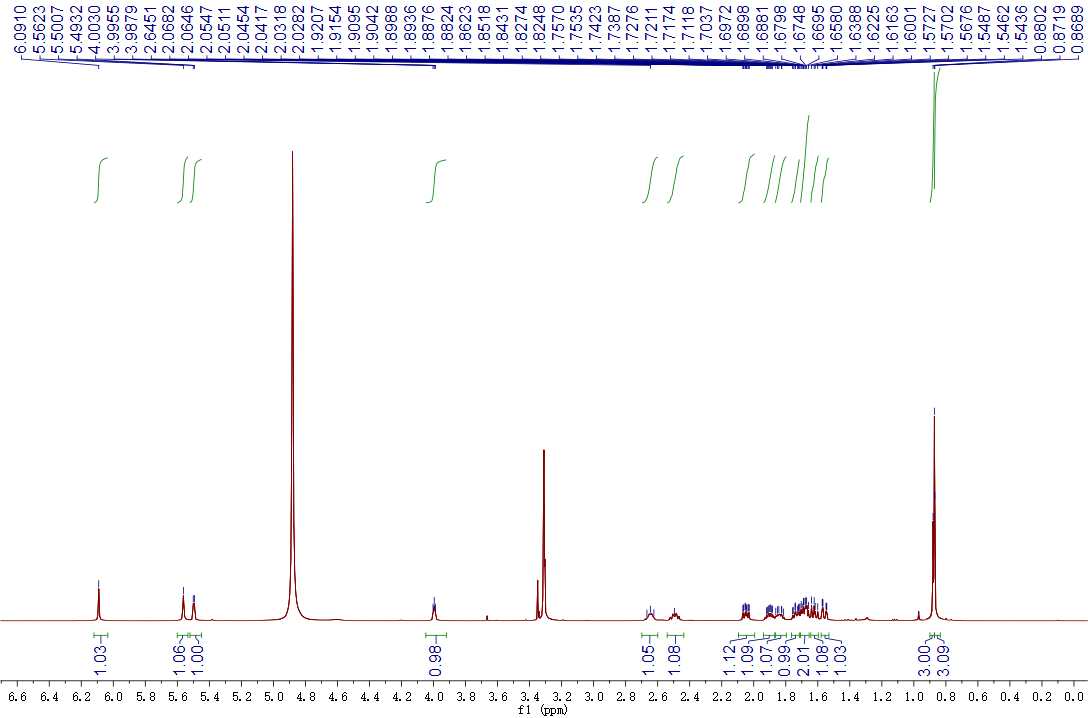


**Figure S1** ^1^H NMR spectrum of laggeranine A in Methanol-*d*_4_


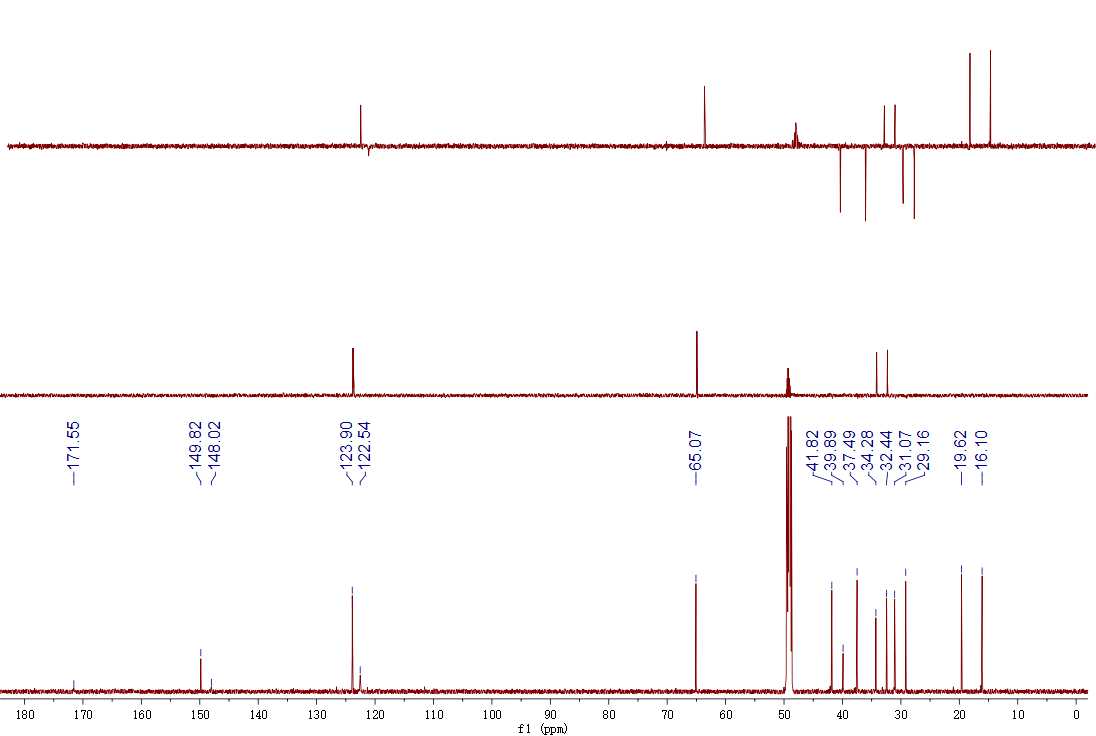


**Figure S2** ^13^C NMR spectrum of laggeranine A in Methanol-*d*_4_


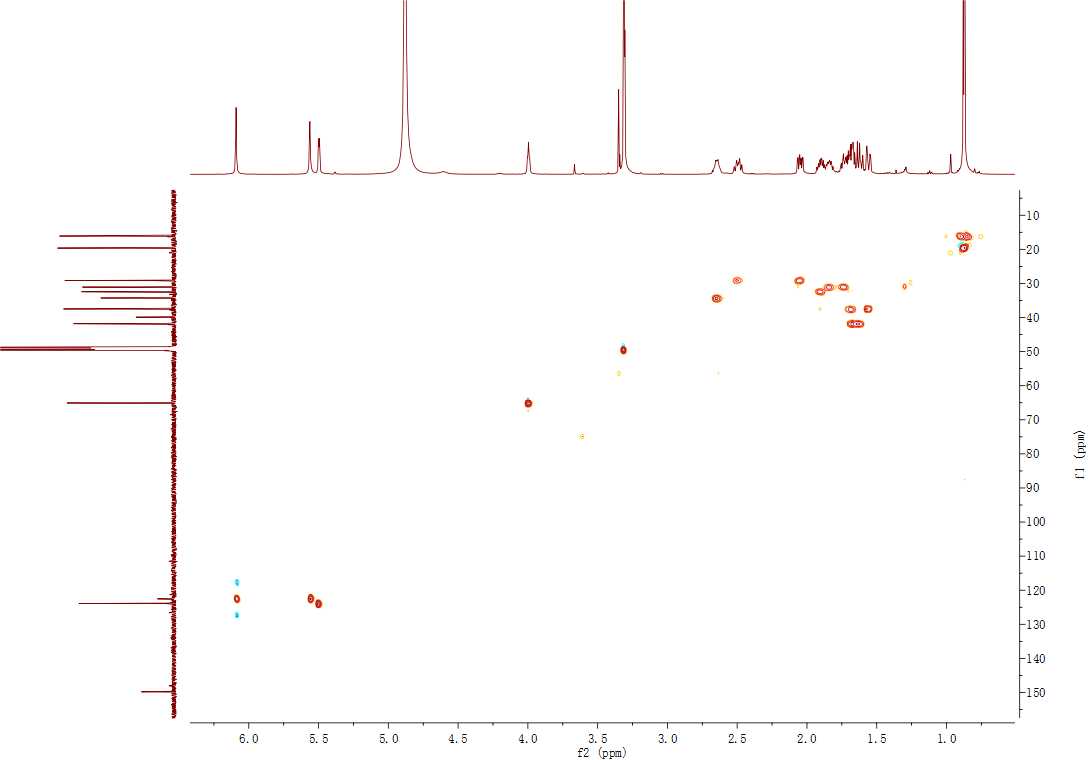


**Figure S3** HSQC spectrum of laggeranine A in Methanol-*d*_4_


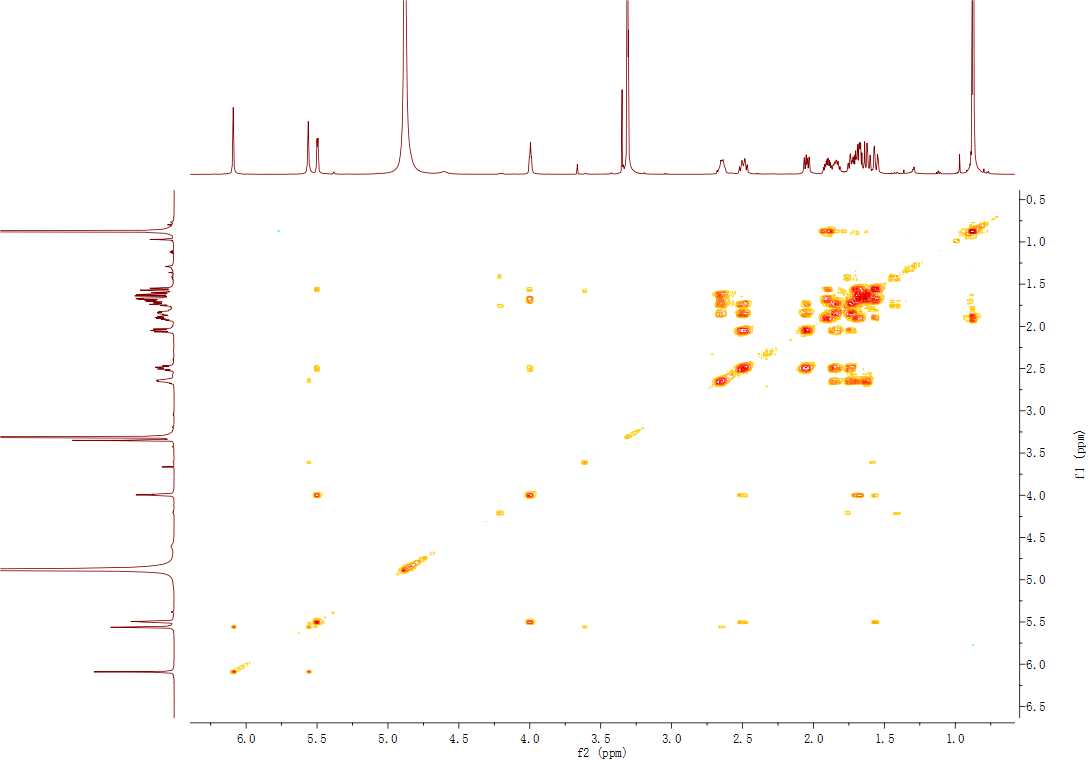


**Figure S4** ^1^H-^1^H COSY spectrum of laggeranine A in Methanol-*d*_4_


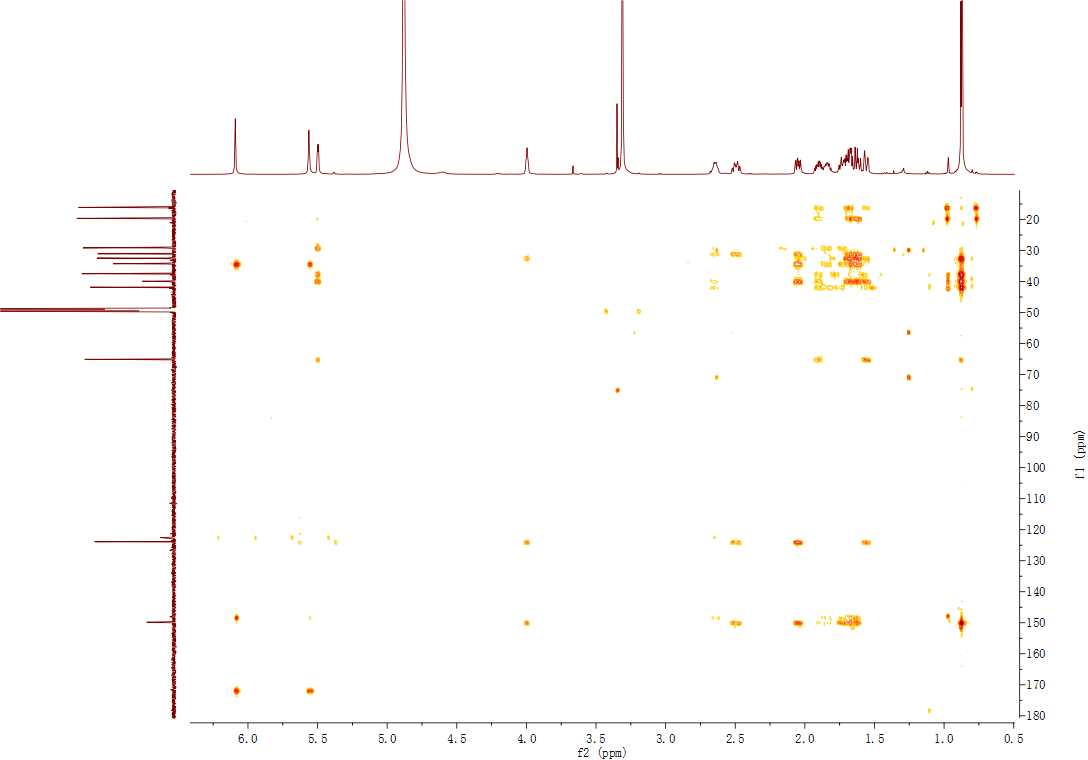


**Figure S5** HMBC spectrum of laggeranine A in Methanol-*d*_4_


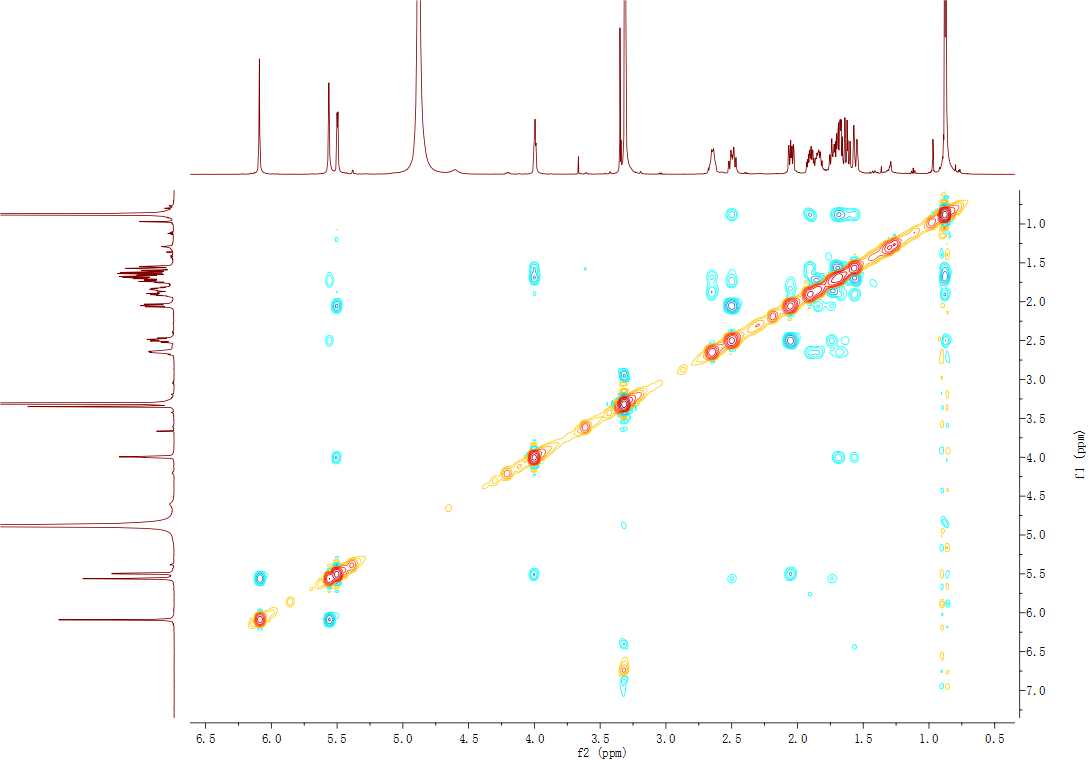


**Figure S6** ROESY spectrum of laggeranine A in Methanol-*d*_4_


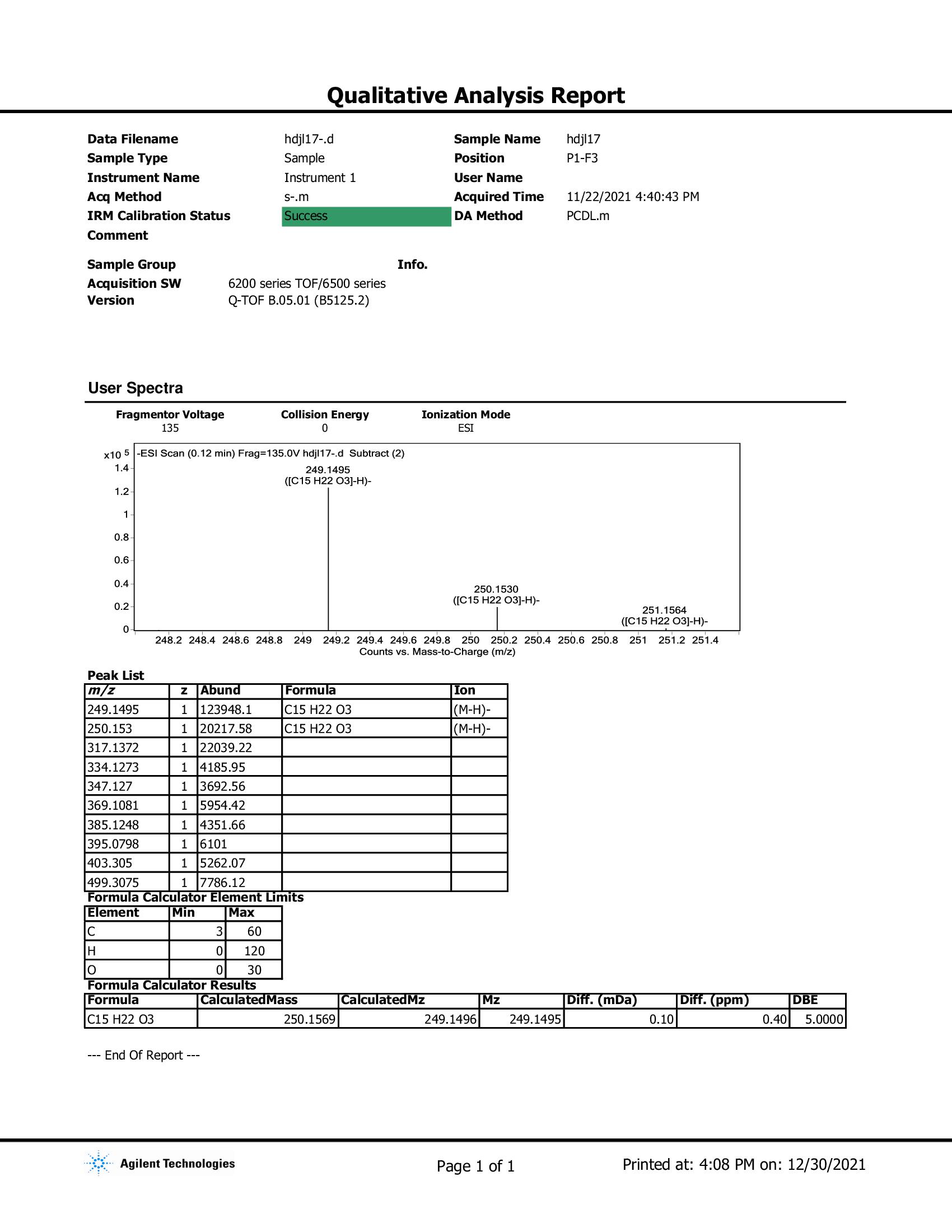


**Figure S7** HRESIMS spectrum of laggeranine A

**Figure S8** IR spectrum of laggeranine A


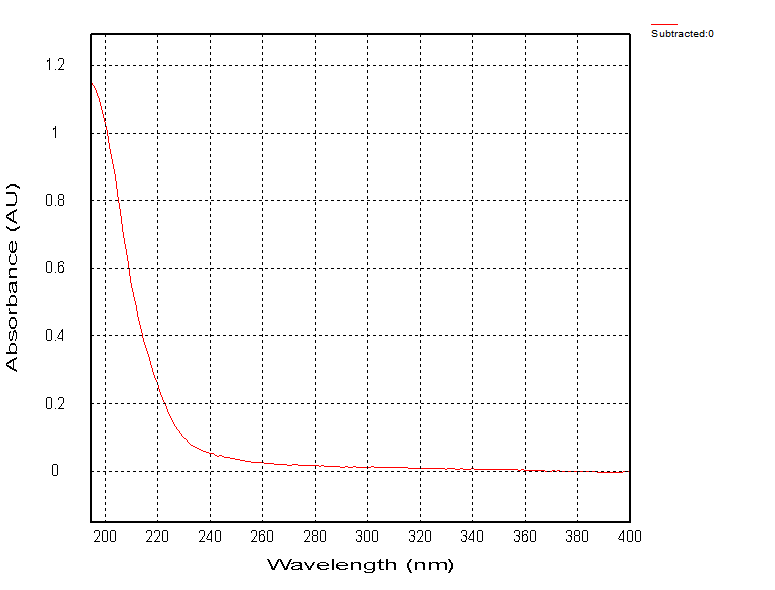


**Figure S9** UV spectrum of laggeranine A


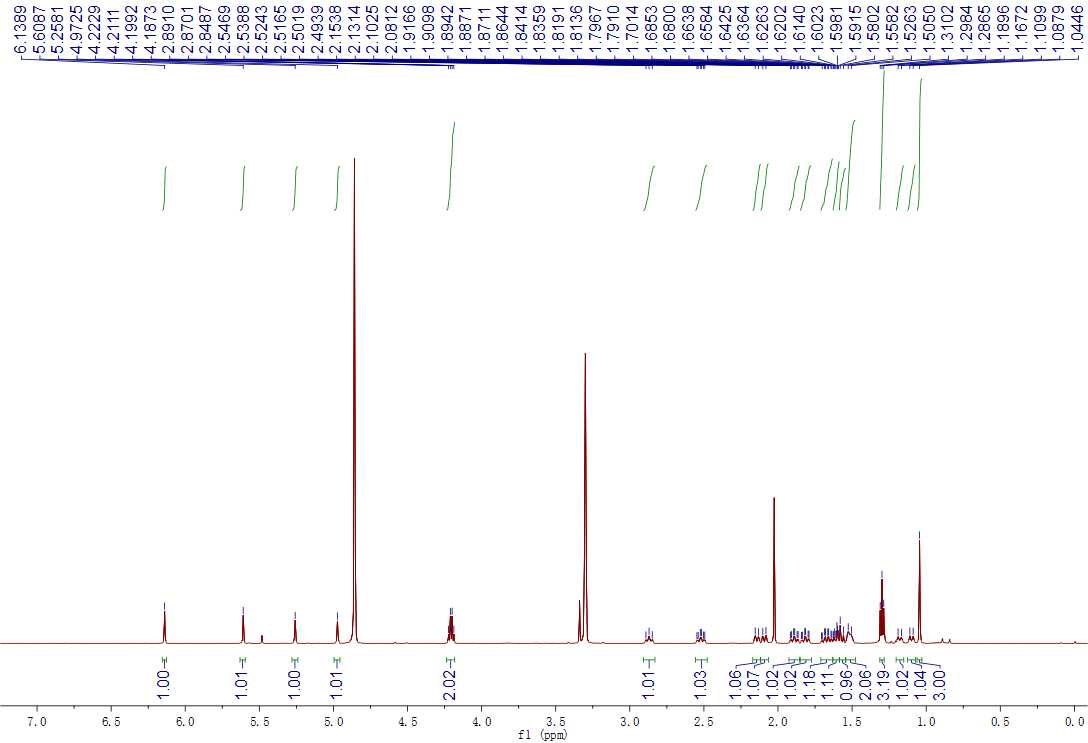


**Figure S10** ^1^H NMR spectrum of laggeranine B in Methanol-*d*_4_


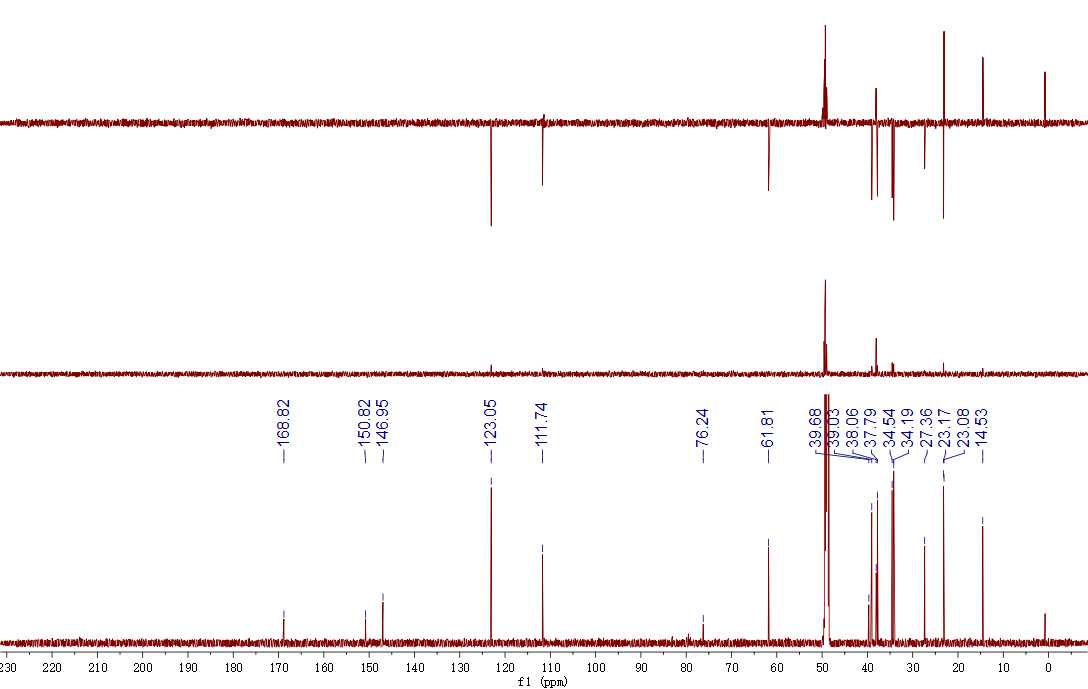


**Figure S11** ^13^C NMR spectrum of laggeranine B in Methanol-*d*_4_


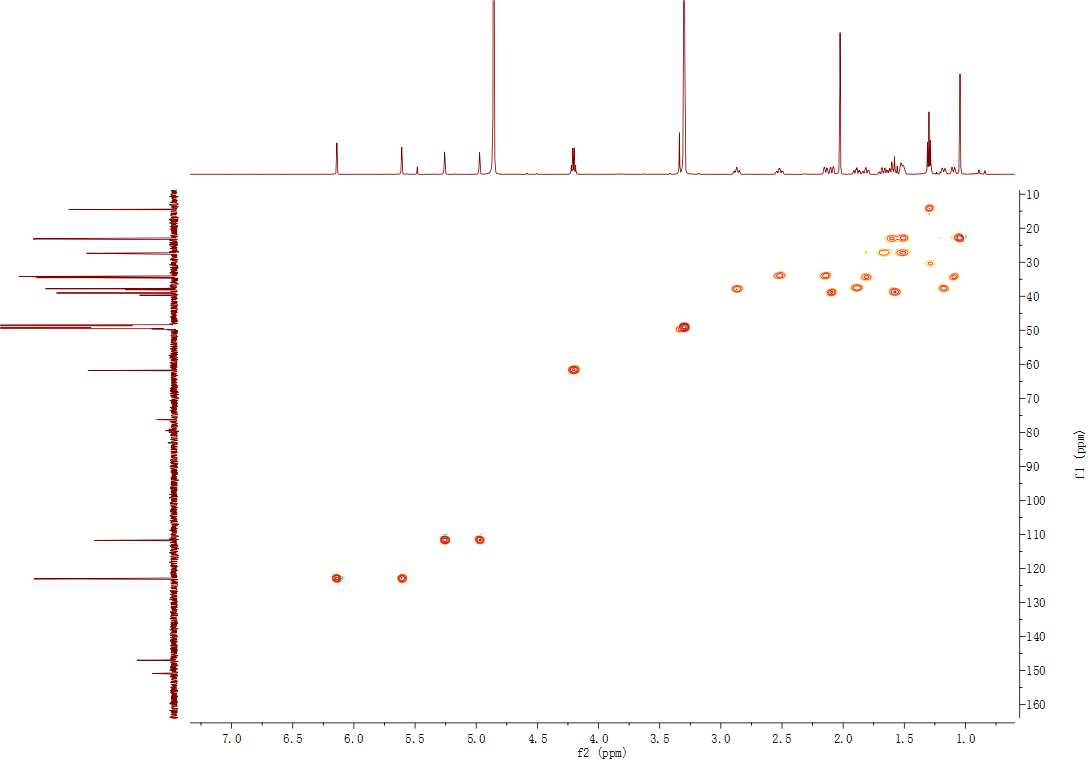


**Figure S12** HSQC spectrum of laggeranine B in Methanol-*d*_4_


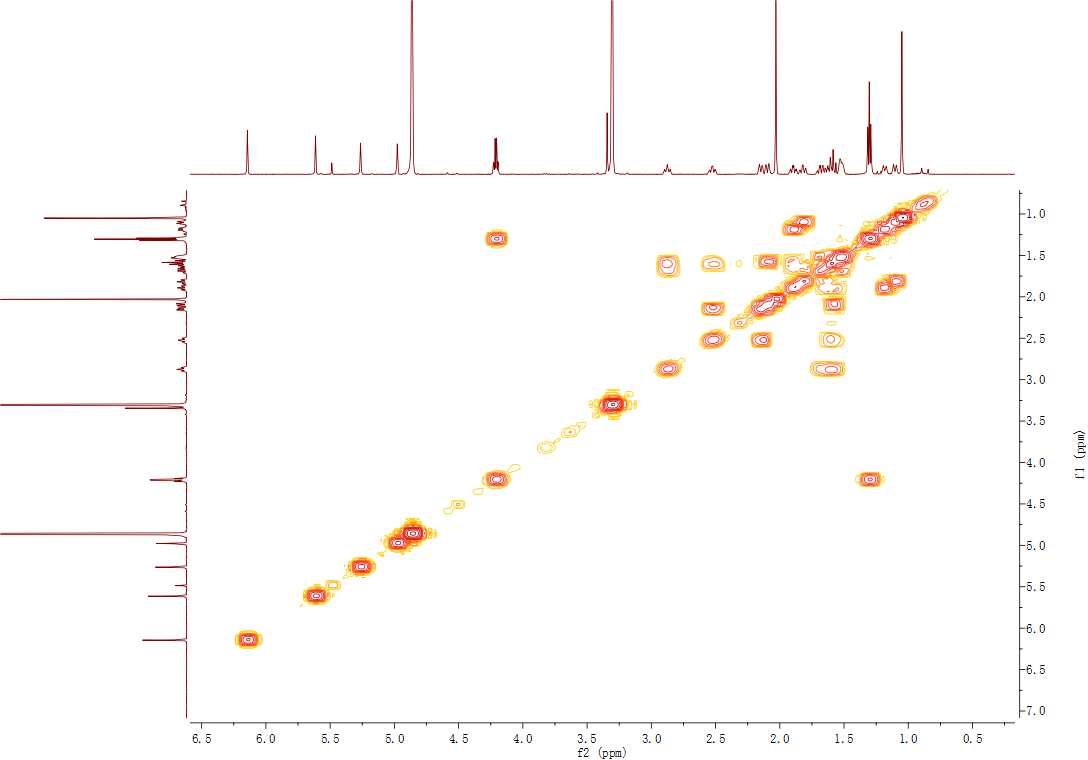


**Figure S13** ^1^H-^1^H COSY spectrum of laggeranine B in Methanol-*d*_4_


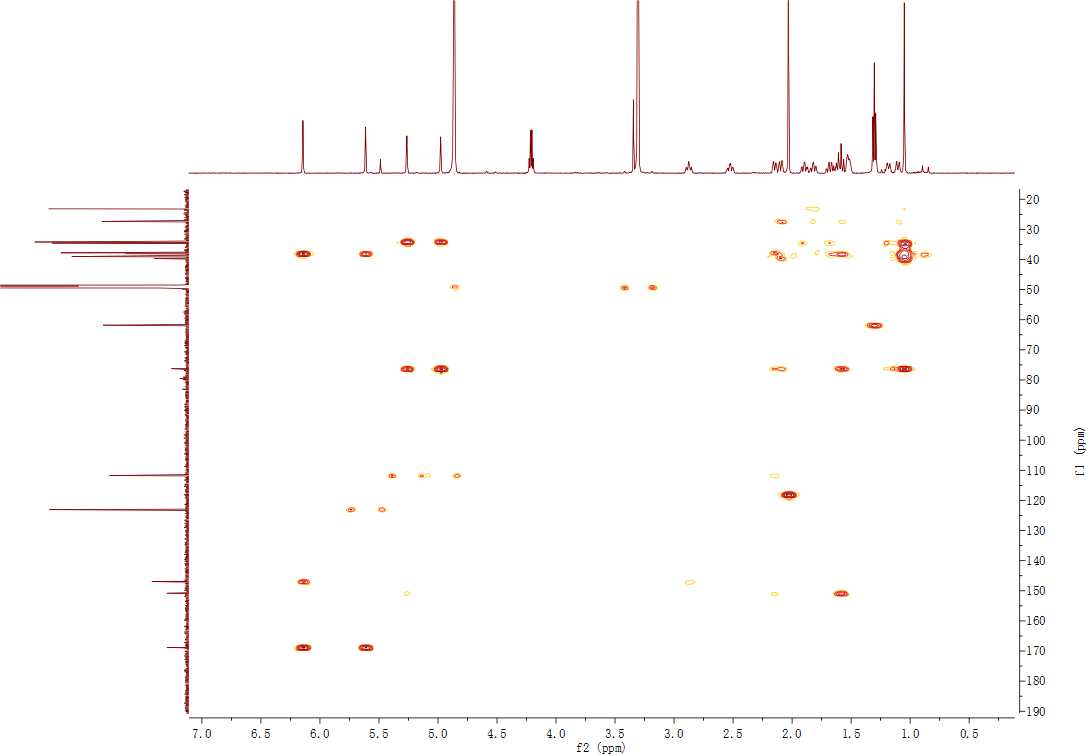


**Figure S14** HMBC spectrum of laggeranine B in Methanol- *d*_4_


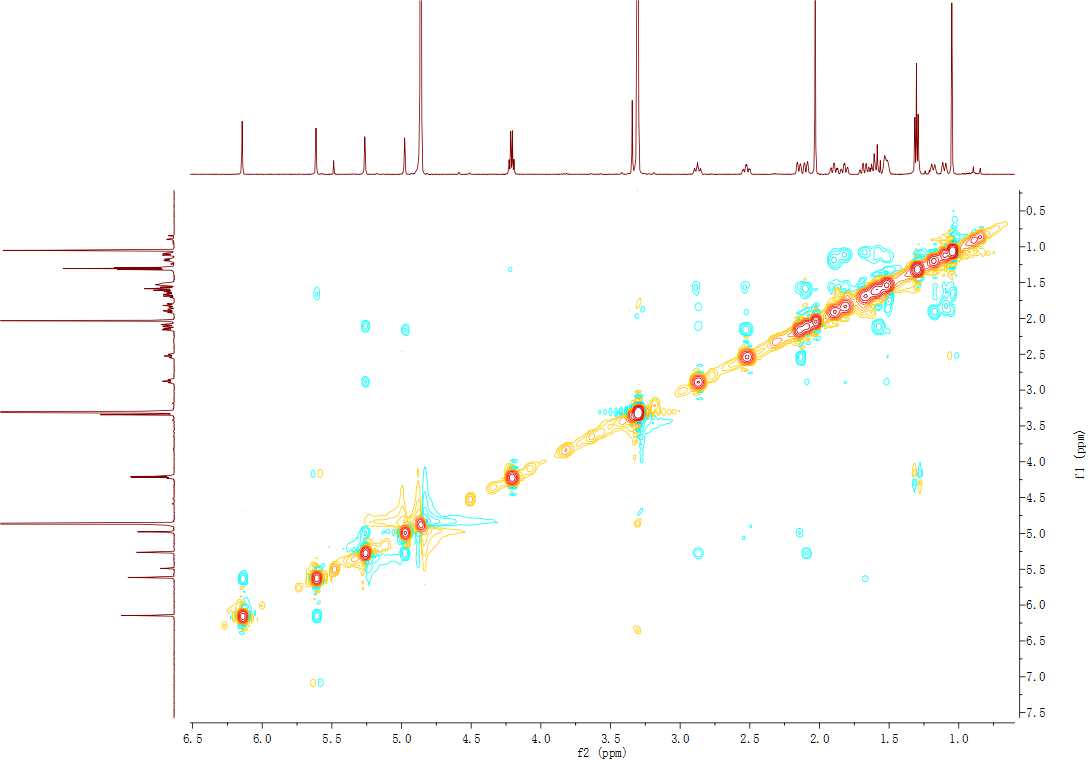


**Figure S15** ROESY spectrum of laggeranine B in Methanol- *d*_4_


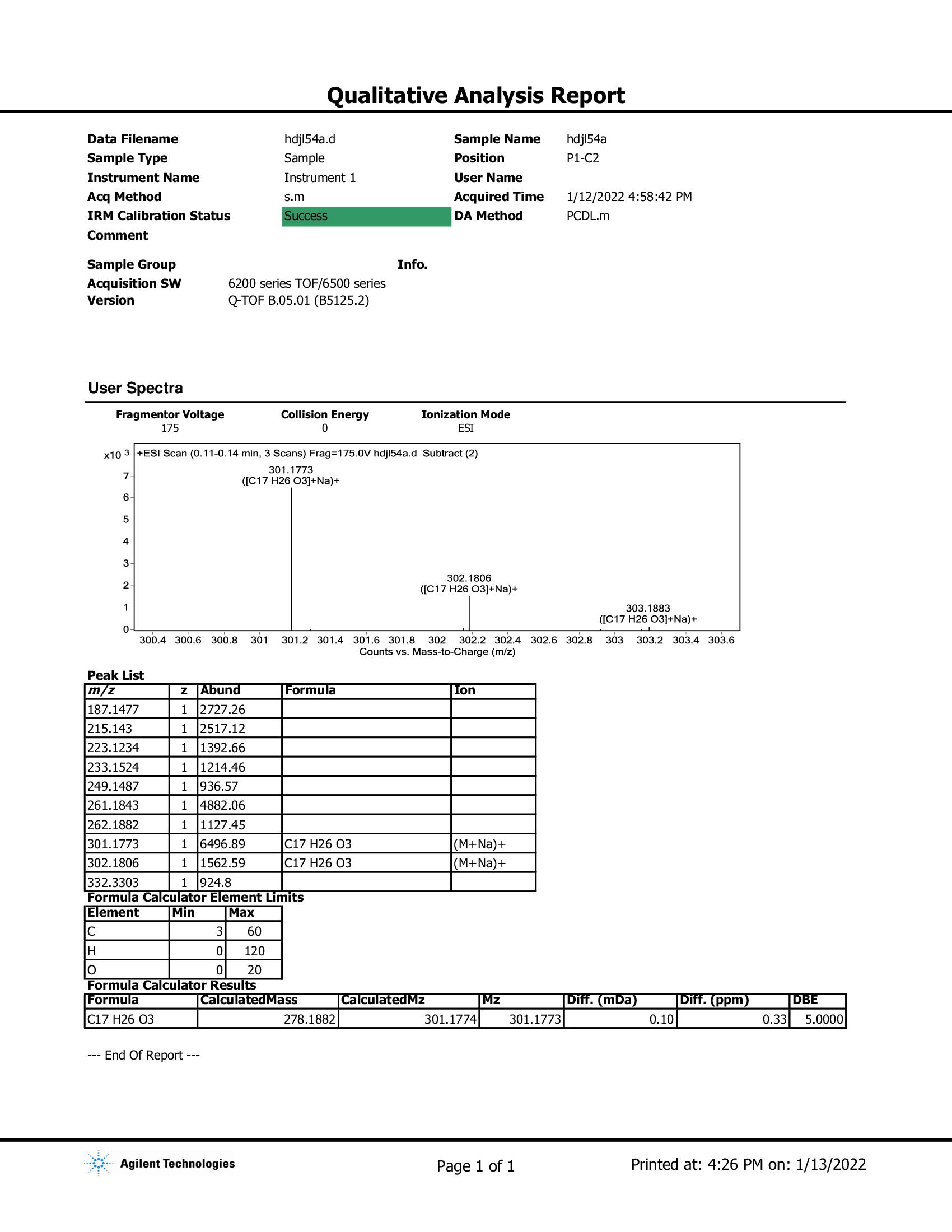


**Figure S16** HRESIMS spectrum of laggeranine B

**Figure S17** IR spectrum of laggeranine B


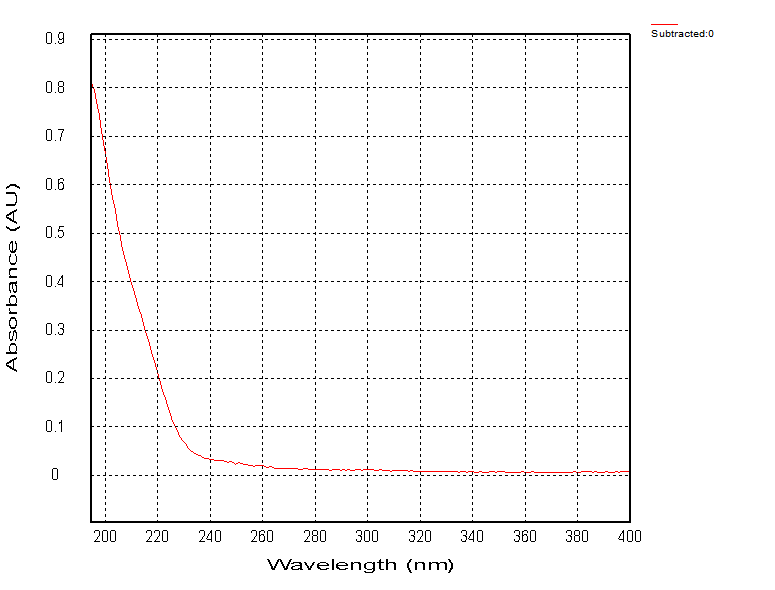


**Figure S18** UV spectrum of laggeranine B


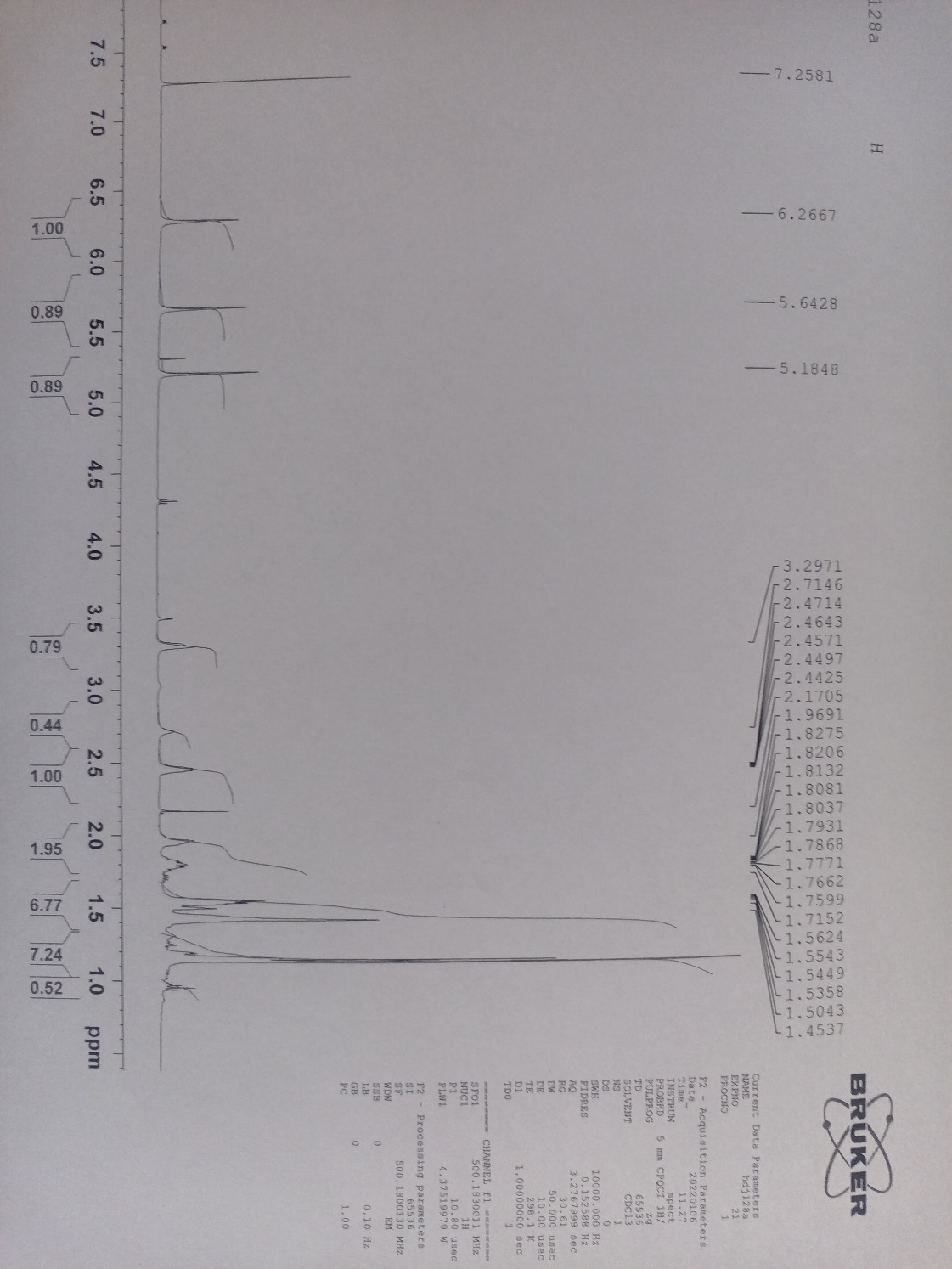


**Figure S19** ^1^H NMR spectrum of compound **3** A in CDCl_3_


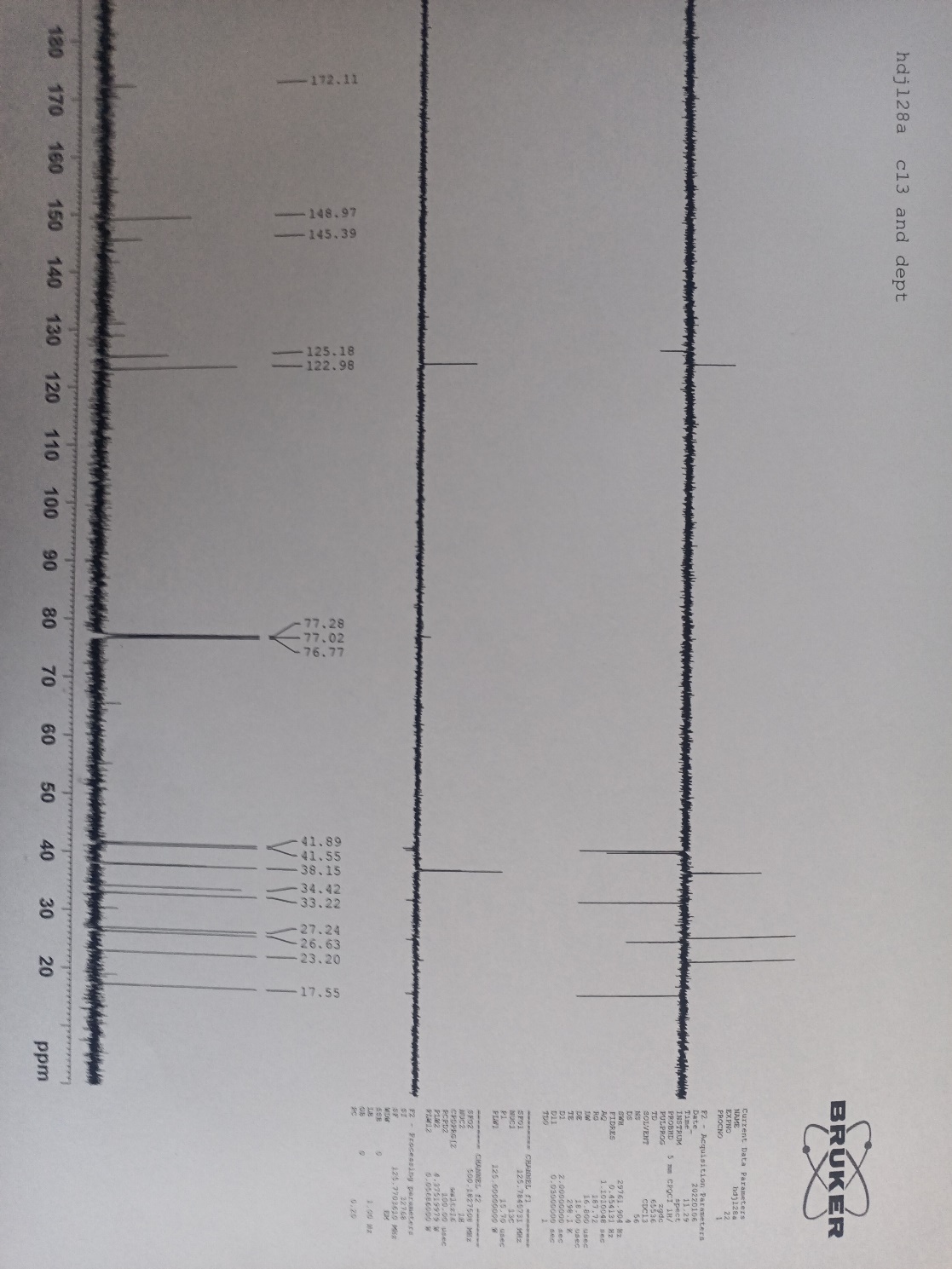


**Figure S20** ^13^C NMR spectrum of compound **3** in CDCl_3_


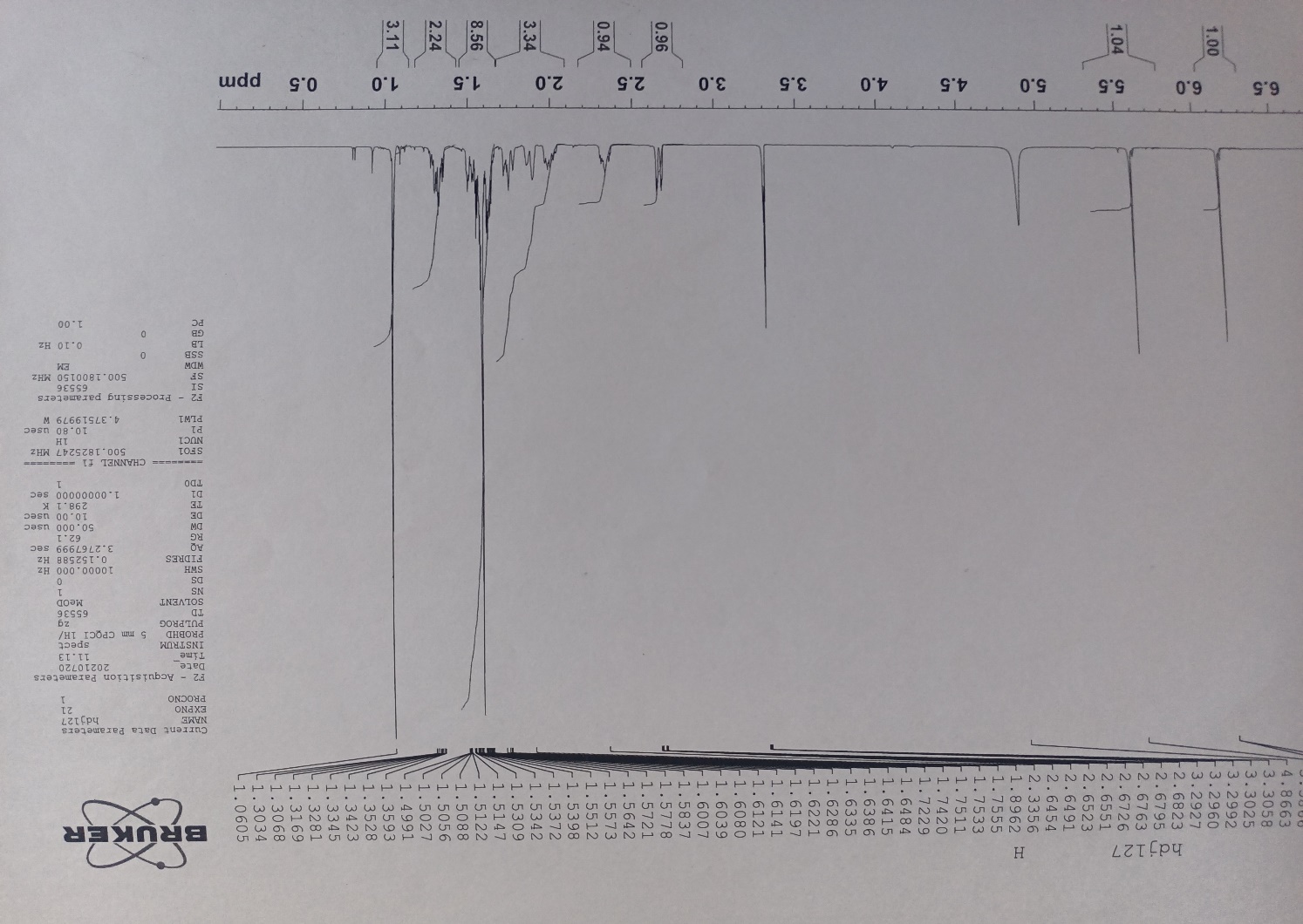


**Figure S21** ^1^H NMR spectrum of compound **4** in Methanol-*d*_4_


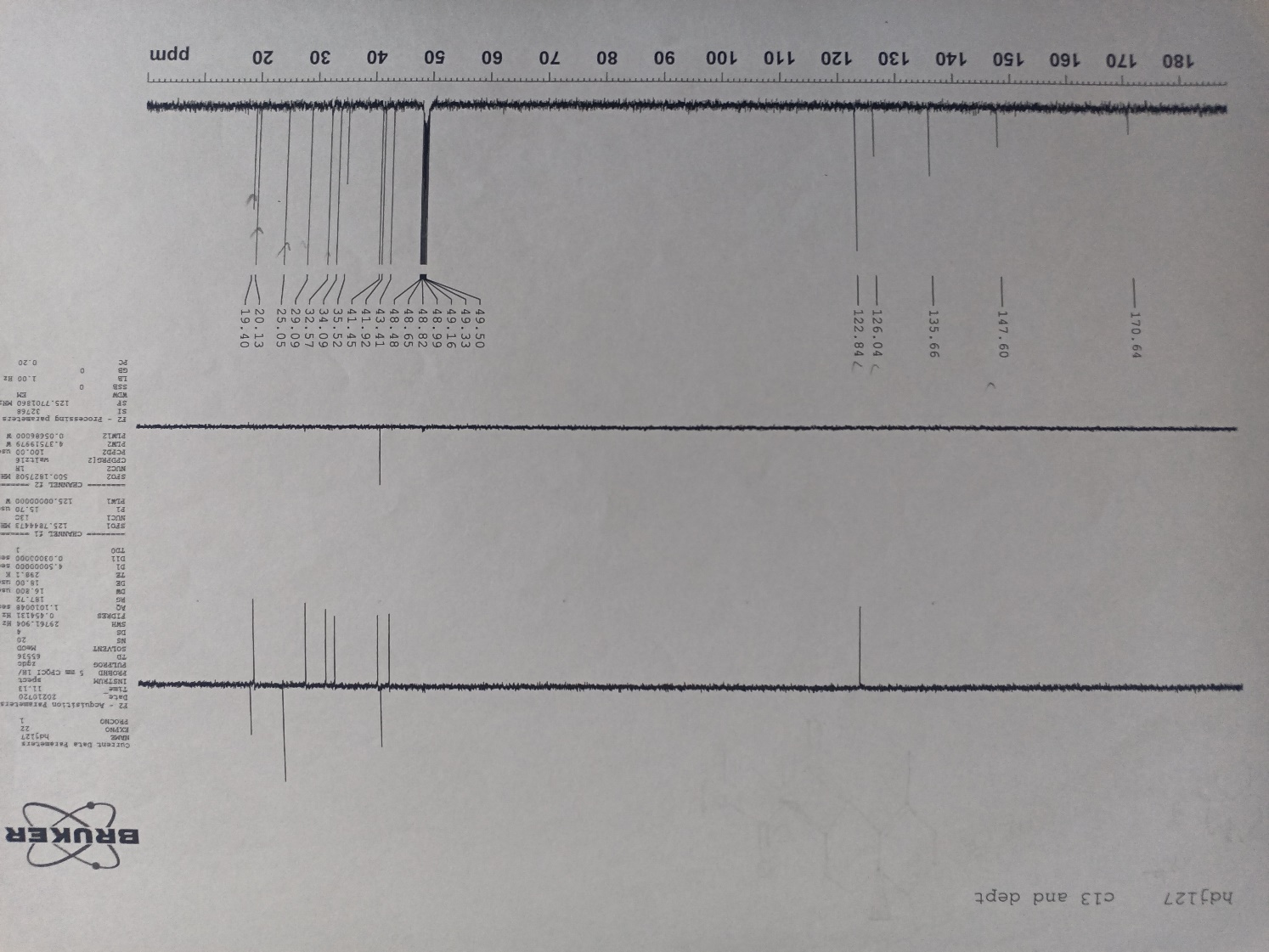


**Figure S22** ^13^C NMR spectrum of compound **4** in Methanol-*d*_4_


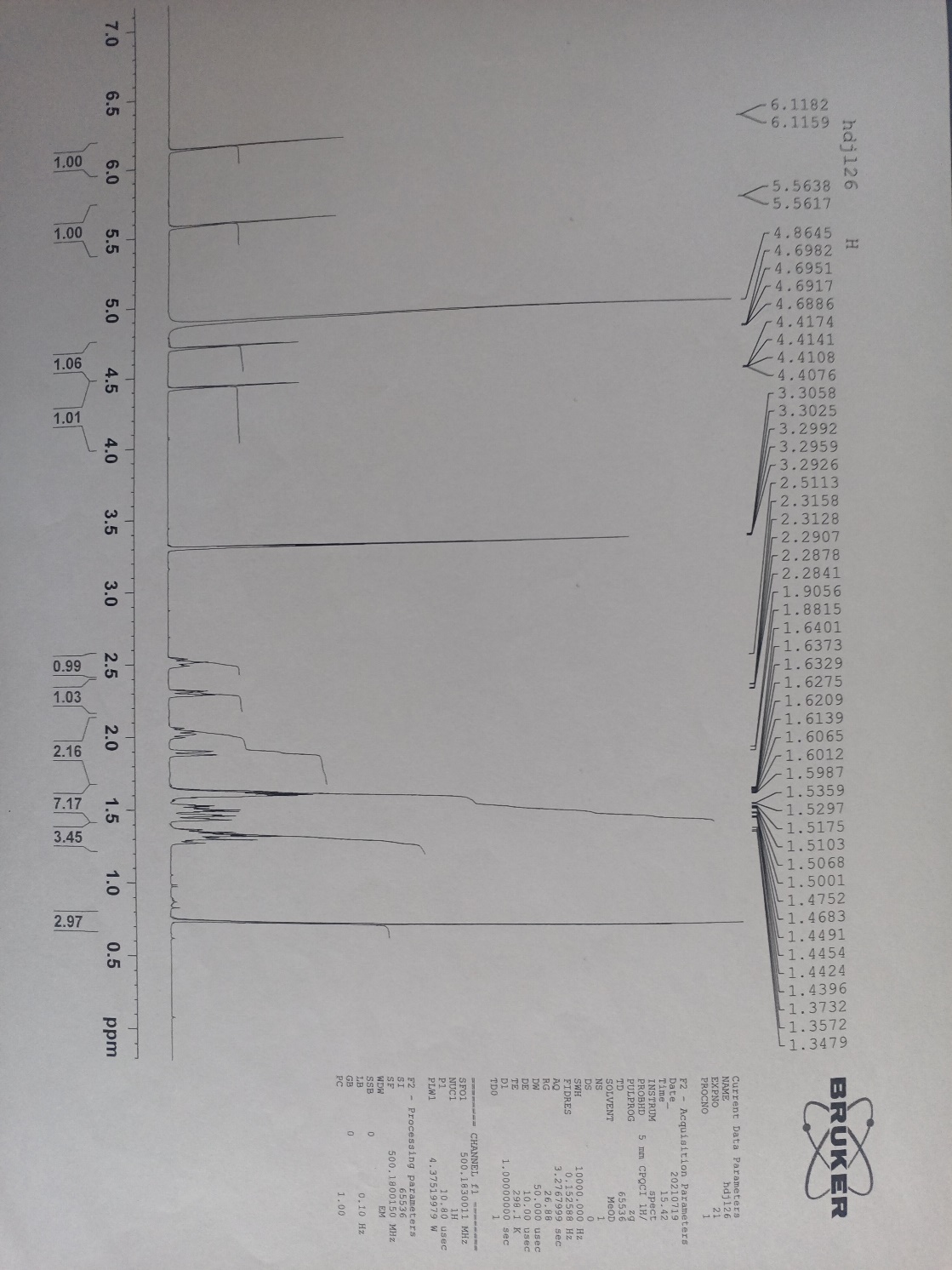


**Figure S23** ^1^H NMR spectrum of compound **5** in Methanol-*d*_4_


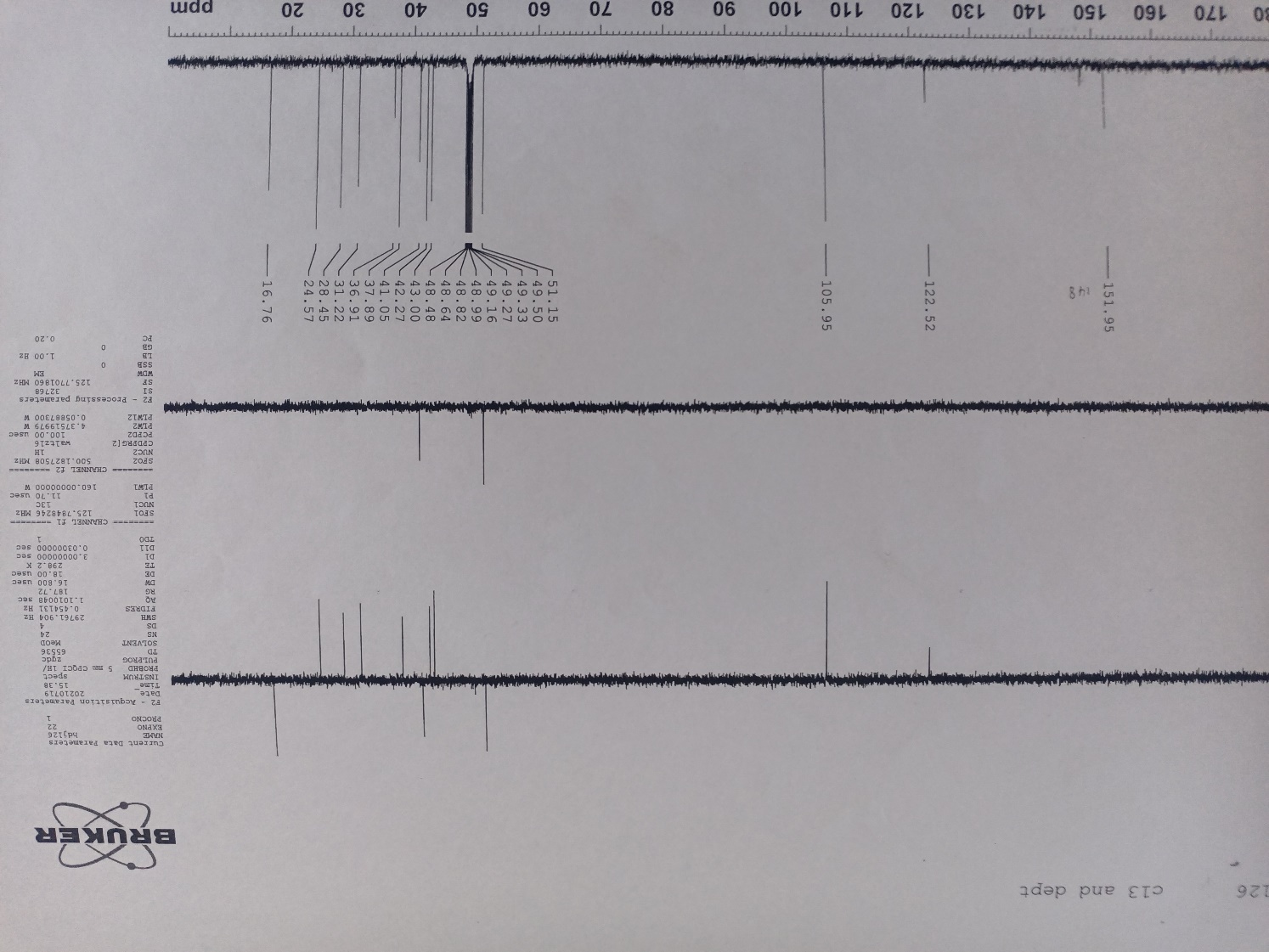


**Figure S24** ^13^C NMR spectrum of compound **5** in Methanol-*d*_4_


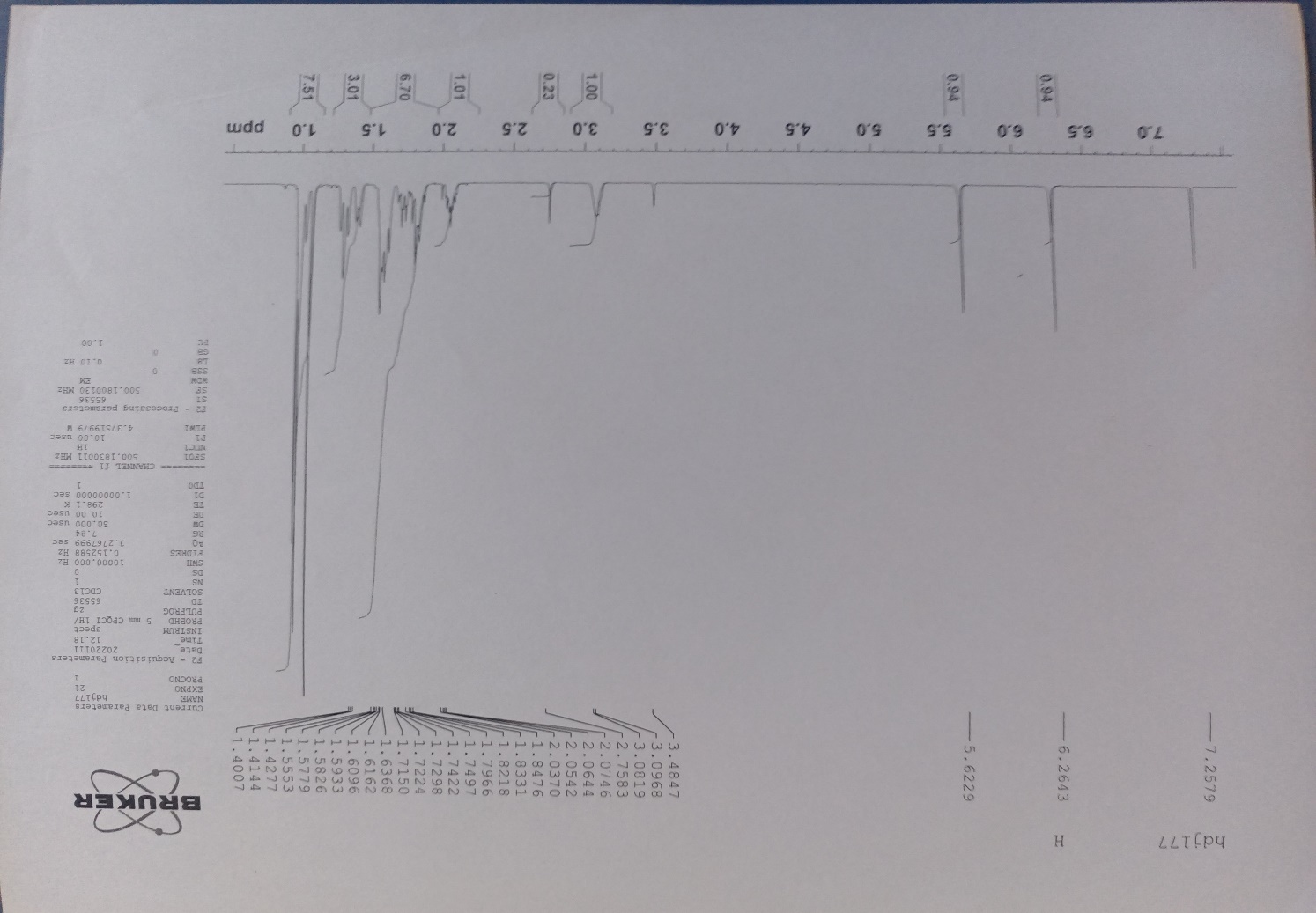


**Figure S25** ^1^H NMR spectrum of compound **6** in CDCl_3_


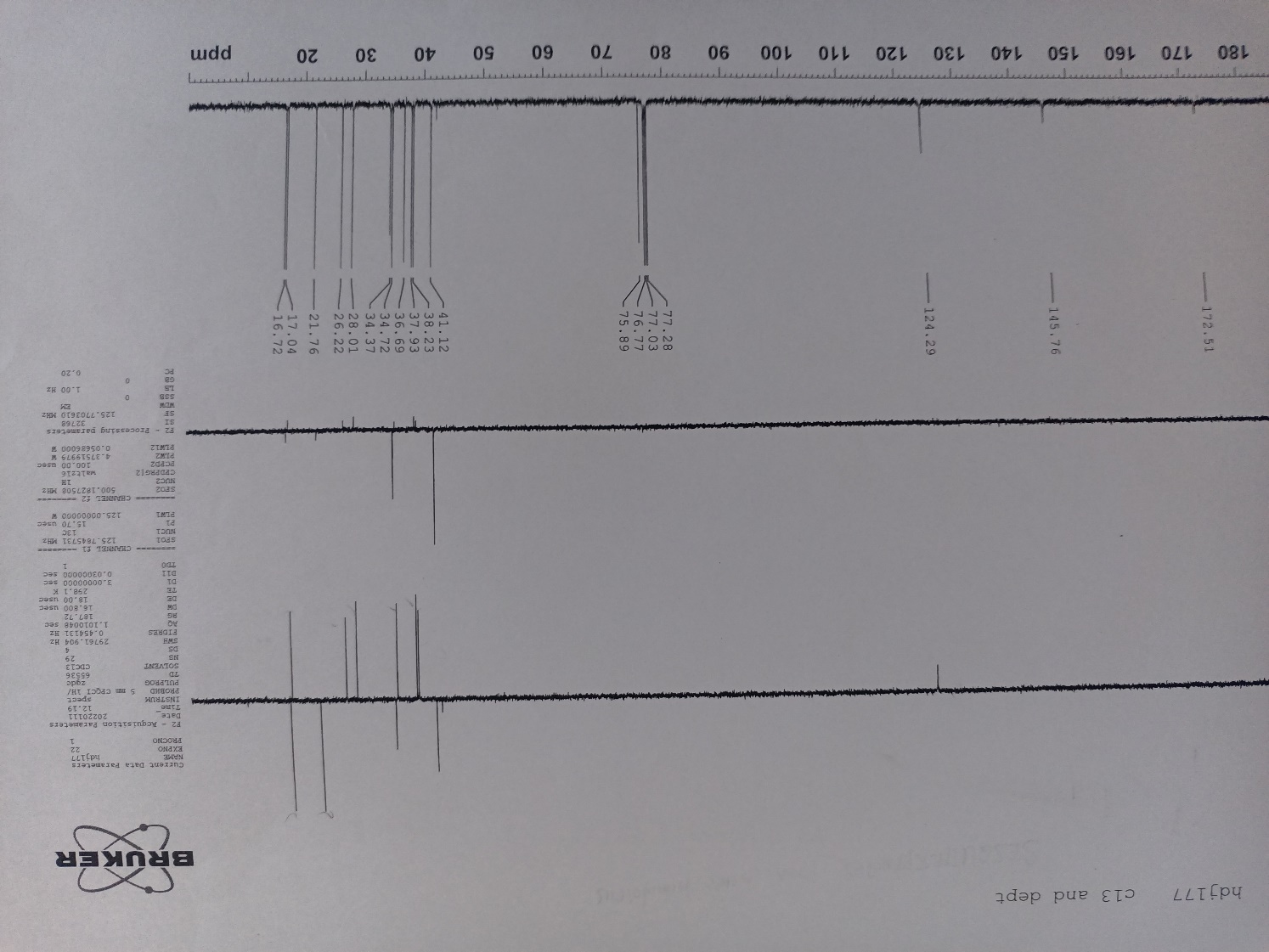


**Figure S26** ^13^C NMR spectrum of compound **6** in CDCl_3_


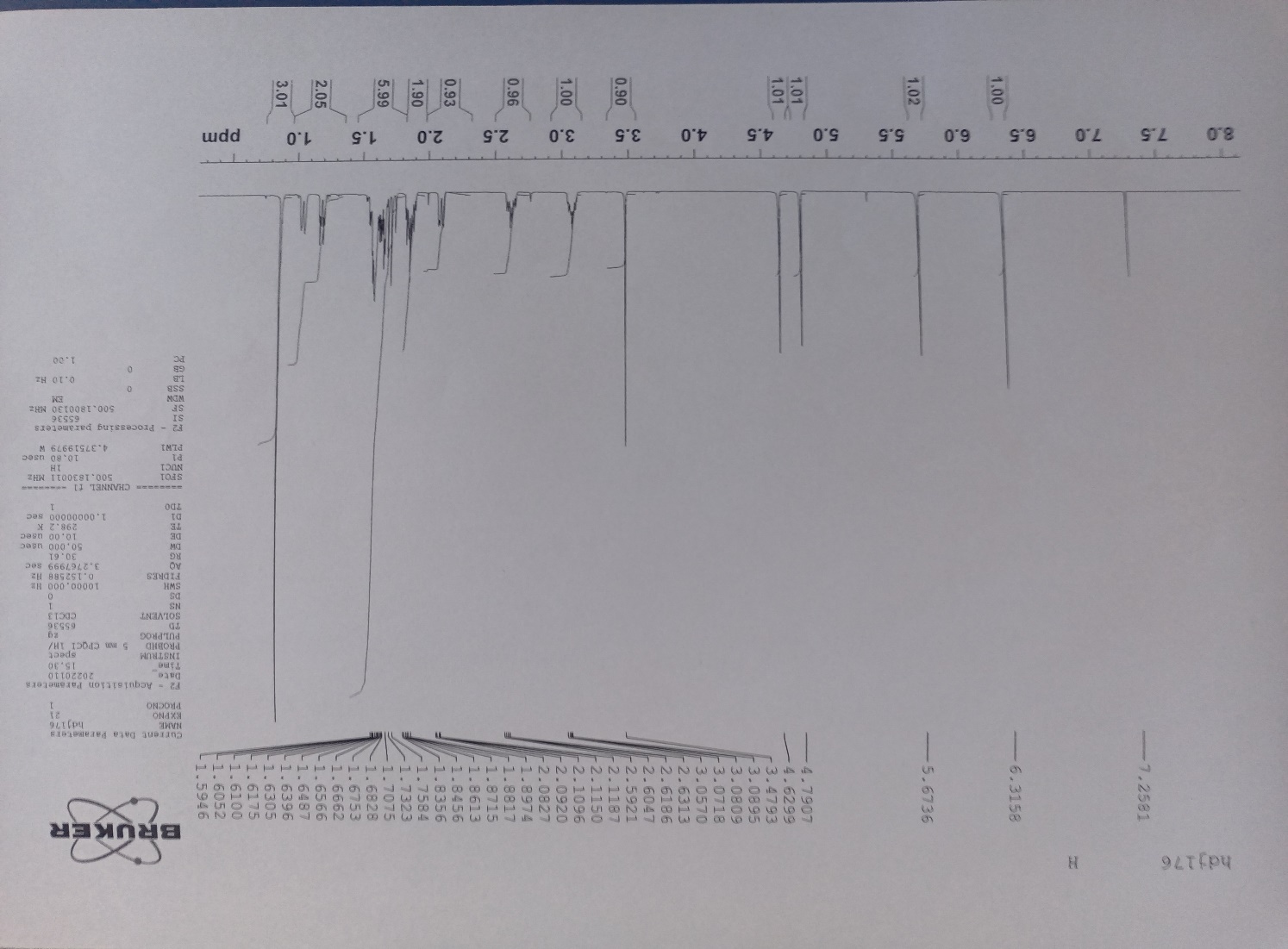


**Figure S27** ^1^H NMR spectrum of compound **7** in CDCl_3_


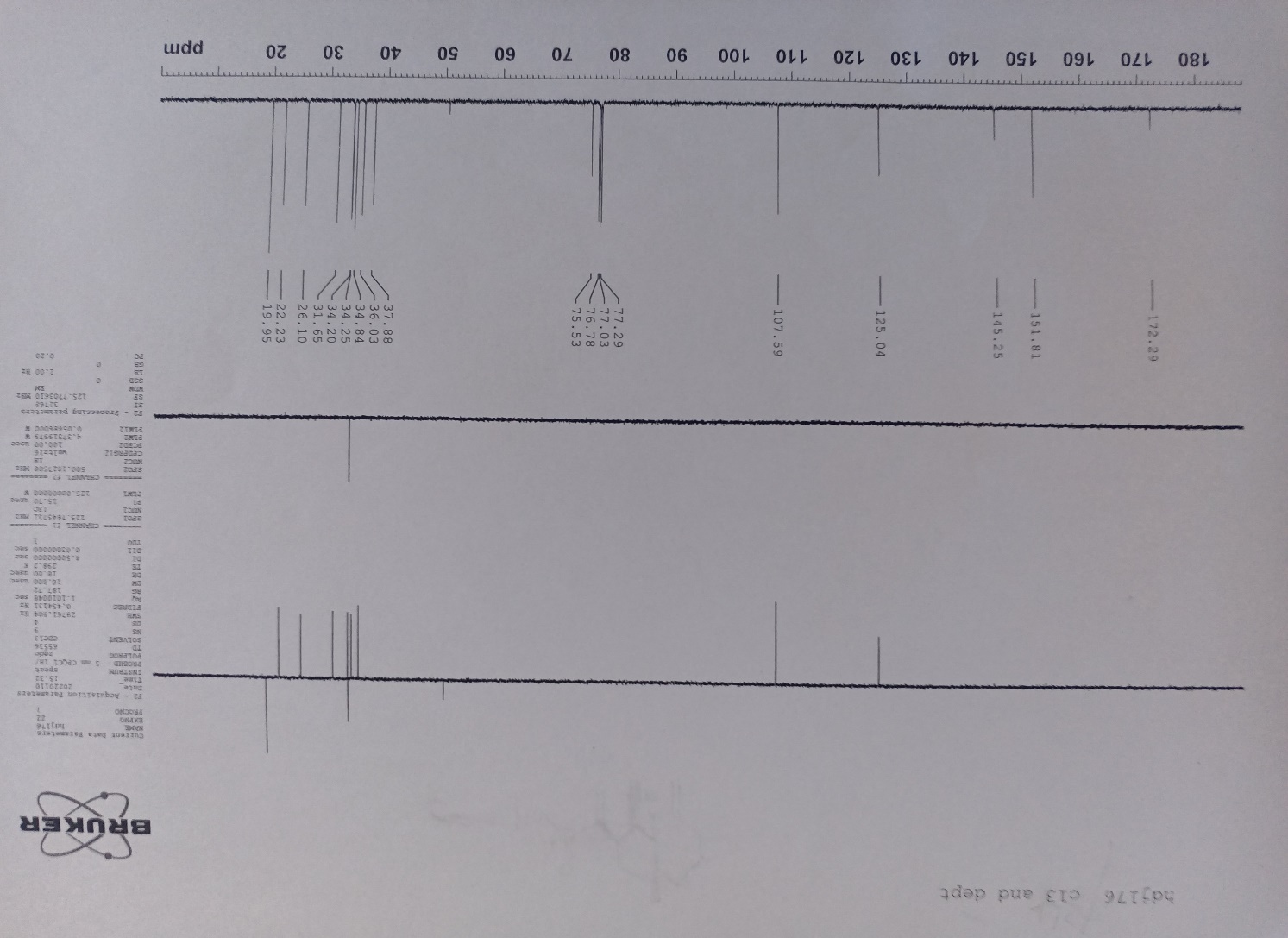


**Figure S28** ^13^C NMR spectrum of compound **7** in CDCl_3_


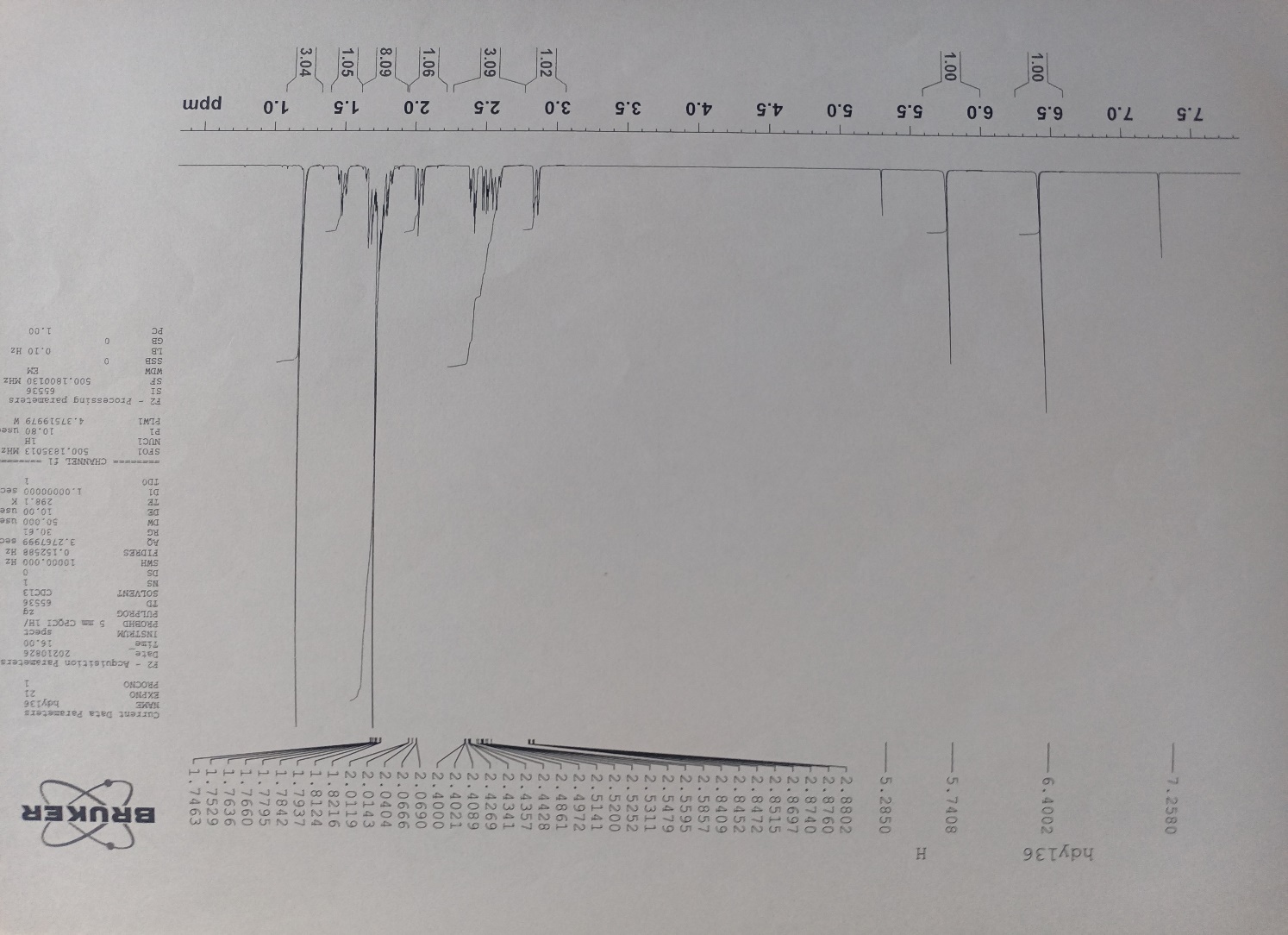


**Figure S29** ^1^H NMR spectrum of compound **8** in CDCl_3_


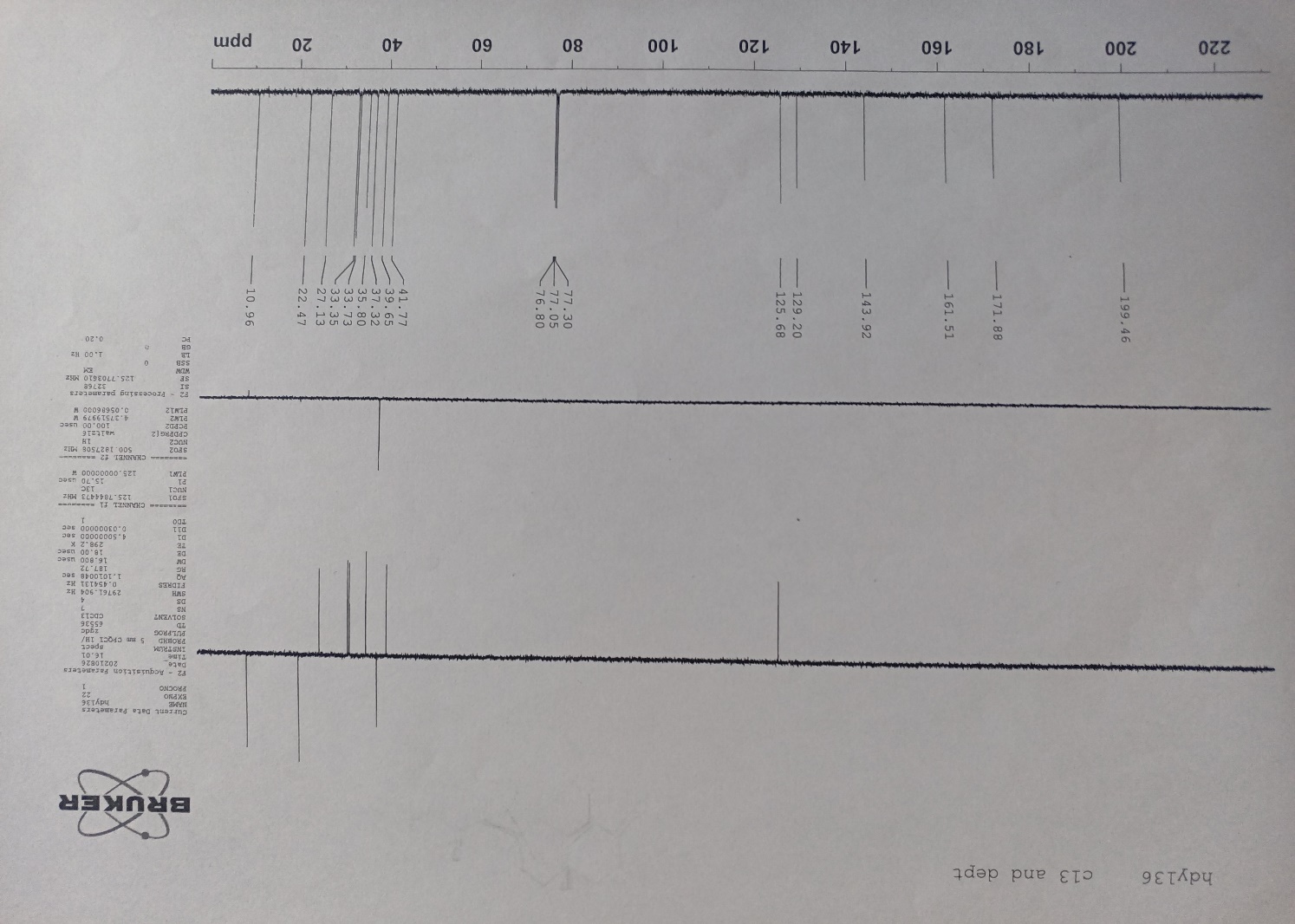


**Figure S30** ^13^C NMR spectrum of compound **8** in CDCl_3_
